# Divergent effects of semaglutide and CRV431 Co-therapy on liver fibrosis and HCC in MASLD mouse model

**DOI:** 10.1371/journal.pone.0358225

**Published:** 2026-09-18

**Authors:** Asha Z. Goodman, Winston T. Stauffer, Philippe A. Gallay

**Affiliations:** Department of Immunology & Microbiology, Scripps Research, La Jolla, California, United States of America; University of Rajshahi, BANGLADESH

## Abstract

Metabolic dysfunction-associated steatotic liver disease (MASLD) is characterized by progressive fibrosis and an increased risk of hepatocellular carcinoma (HCC). Recently, approved drug treatments for MASLD, including thyroid hormone receptor beta (THR-β) and glucagon-like peptide-1 (GLP-1) receptor agonists, have been effective in treating the underlying metabolic causes of MASLD. However, more effective and acute treatments, particularly for already advanced or cirrhotic steatohepatitis, remain elusive. Other drug candidates, such as cyclophilin inhibitors, which have shown promise for treating advanced MASLD, are still under investigation. Because advanced MASLD reflects both upstream metabolic stress and downstream, self-sustaining injury responses promoting inflammation and fibrogenesis, we investigated whether pairing GLP-1 agonism (metabolic correction) with cyclophilin inhibition (fibrosis and inflammation remodeling) would yield additive or emergent benefits. Thus, we evaluated Semaglutide, Rencofilstat (CRV431), and their co-administration in a C57BL/6J model of diet- and toxin-induced MASLD via a Western diet, sucrose supplementation, and chronic carbon tetrachloride exposure. Semaglutide monotherapy significantly reduced body weight and decreased HCC burden (Kruskal-Wallis Analysis, *p* < 0.0001) whereas fibrosis quantified by picrosirius red staining did not differ significantly across the treatment groups (Welch’s Analysis of Variance, *p* = 0.1725). Co-therapy did not improve collagen deposition compared with monotherapy. These findings indicate that the combination of GLP-1 receptor agonism with cyclophilin inhibition does not confer additive antifibrotic benefits in this advanced MASLD model, underscoring stage-dependent constraints on therapeutic synergy.

## Introduction

Recent advances in the management of metabolic diseases have accelerated the clinical adoption of glucagon-like peptide-1 receptor agonists (GLP-1 RAs), including Semaglutide. Originally formulated for the treatment of type 2 diabetes and subsequently leveraged for chronic weight management, Wegovy (Semaglutide) received FDA approval on March 2025 for adults with noncirrhotic metabolic dysfunction-associated steato-hepatitis (MASH) and moderate-to-advanced fibrosis (FDA Wegovy label, 2025), expanding interests in GLP-1-based combination strategies for metabolic dysfunction-associated steatotic liver disease (MASLD; [1]. Approximately one-third of adults worldwide are diagnosed with MASLD [2]. While the onset of MASLD is initiated by dietary excess cited as high-fat consumption, the progression of the disease is marked by hepatic collagen deposition and fibrotic scarring, as the liver attempts to counteract chronic levels of inflammation and dysregulated lipogenesis. GLP-1 receptor agonists, such as Semaglutide, act primarily through central appetite regulation and delayed gastric emptying in addition to enhancing insulin secretion, reducing hepatic de novo lipogenesis, and improving mitochondrial function in hepatocytes [3,4].

The optimization of the treatment of advanced fibrosis in progressive MASLD has been driven by emerging research on CRV431 (Rencofilstat; [5], a non-immunosuppressive cyclophilin inhibitor, and a Cyclosporin A analog that inhibits cyclophilin peptidyl-prolyl isomerase activity by binding to the hydrophobic and enzymatic pocket of cyclophilins. Deletion of various cyclophilin family members or their inhibition by treatment with CRV431 significantly ameliorated advanced MASLD-induced liver fibrosis and the appearance and progression of HCC nodules [6,7]. Cyclophilin inhibition represents a mechanistically distinct strategy for targeting fibrogenic remodeling as multiple cyclophilin isoforms, including cyclophilin A and cyclophilin B, have been implicated in pro-fibrotic signaling (e.g., TGF-β-associated pathways), extracellular matrix production and maturation, inflammatory amplification, and oncogenic processes across visceral organs [6,8,9] Cyclophilin D plays a distinct role relative to other cyclophilin isoforms in the progression of MASLD-related HCC [10,11].

Here, we evaluated Semaglutide, CRV431, and their combination in a murine model of MASLD induced by the Friedman method, employing a two-hit strategy of *ad libitum* [12] and carbon tetrachloride-induced hepatotoxicity injury. This model recapitulates the key features of advanced human disease, including steatosis, lobular inflammation, bridging fibrosis, and spontaneous tumorigenic phenotypes consistent with hepatocellular carcinoma (HCC). We hypothesized that targeting systemic metabolic dysfunction via GLP-1 receptor activation and intrahepatic fibrogenic signaling via cyclophilin inhibition would yield additive or synergistic improvements in fibrotic regression and the attenuation of hepatocarcinogenic outcomes.

## Materials and methods

**Fig 1:**
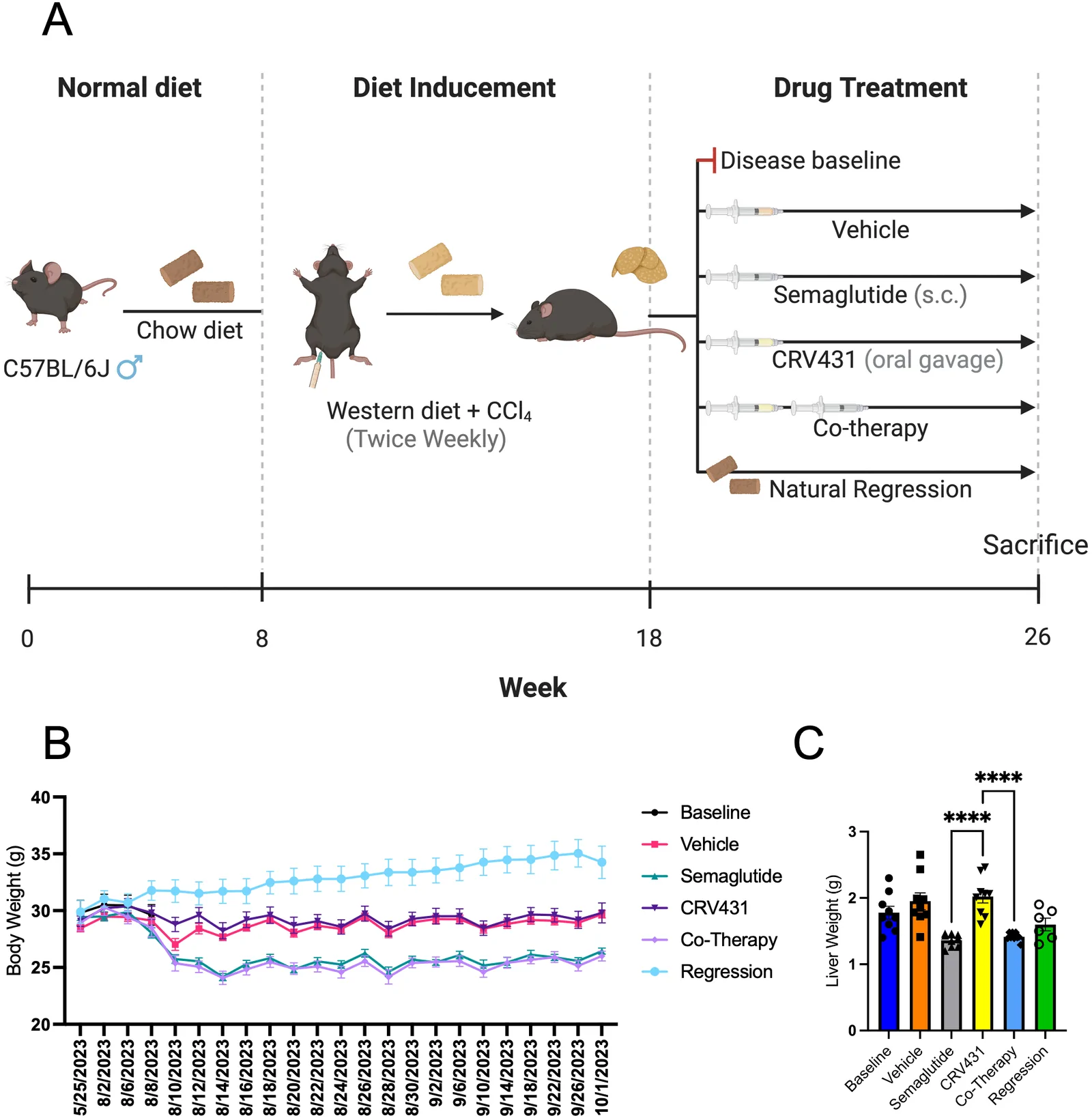
Study design and effect of treatment on body and liver weight. (A) Schematic of the experimental design, treatment groups, and study timeline (created with BioRender.com**.** (B) Mouse body weight (g) recorded across treatment duration. Data are shown as mean ± SEM. (C) Terminal liver weights measured at time of organ collection, reported as absolute organ weight.

In this study, male C57BL/6J mice (n = 54 total, n = 9 per group) were used as a preclinical model to recapitulate key pathological features of MASLD observed in human disease. Male mice were used exclusively because the Western diet plus carbon tetrachloride (CCl_4_) two hit model produces more uniform and penetrant fibrosis and hepatocellular carcinoma in males, whereas female mice show slower and more variable progression in high fat, MASH driven models, in part reflecting the hepatoprotective effects of estrogen and the estrogen dependent suppression of IL-6 driven hepatocarcinogenesis; a single sex design therefore reduced variability for the state matches comparison [13,14]. Mice were housed in groups of four to five per cage in individually ventilated cages containing carbon enrichment tubes and maintained on a 12-hour light/dark cycle at 21 °C. Mice were obtained from the dedicated breeding colony in-house. The model used here is a modification of the diet and toxin-induced steatohepatitis model described by Tsuchida and colleagues, in which a Western diet high in fat, fructose, and cholesterol is combined with low dose CCl_4_ to accelerate progression from steatohepatitis through fibrosis to hepatocellular carcinoma [13]. In the original report, male C57BL/6J mice on this regimen developed advanced fibrosis by approximately 12 weeks and hepatocellular carcinoma by approximately 24 weeks and recapitulated the histologic, immunologic, and transcriptomic features of human NASH. To induce advanced MASLD with fibrosis and HCC features, we used a modified version of the Friedman method combining a Western diet (42% kcal from fat, increased sucrose, 1.25% cholesterol; Inotiv TD.120528, Madison, WI, USA) [13] with 10% (w/v) sucrose provided *ad libitum* in drinking water for 10 weeks at 8 weeks of age. Hepatotoxic injury was induced by intraperitoneal injections of CCl_4_ (0.2 mL/kg diluted 1:4 in corn oil; cat. No. 501860020, distributed by Sigma-Aldrich: St. Louis, MO, USA) twice weekly throughout the 10 week induction period [15]. Following the induction period, mice were randomly assigned to 8 weeks of treatment with the drug or vehicle. To assess the regression driven by dietary withdrawal, one group discontinued the Friedman diet and returned to standard chow. An overview of the experimental design, treatment groups, and study timeline is shown in Fig 1A. six groups: Baseline, harvested at the end of the induction period to define pretreatment disease severity; Vehicle; Semaglutide (cat. No. HY-114118, distributed by Fisher Scientific Cat. No. 50-225-9952; Pittsburgh, PA, USA), CRV431 (obtained from Hepion Pharmaceuticals (Edison, NJ)); Co-therapy (CRV431 and Semaglutide treated in tandem) and Regression (in which the Friedman diet was withdrawn, and animals were returned to standard chow during the treatment window. Comparisons of primary interest were each monotherapy and the co-therapy against vehicle, and the co-therapy against each monotherapy. Semaglutide was prepared as concentrated stock aliquots and stored at −80 °C. Working dilutions were prepared fresh weekly in phosphate-buffered saline (PBS) supplemented with 0.1% bovine serum albumin (BSA) to minimize peptide absorption to labware and stored at 4 °C until daily subcutaneous (s.c.) injections were administered. Dosing solutions were prepared at 0.12–6 nmol/mL (0.12–6 µM), depending on titration day and target dose. To minimize gastrointestinal side effects, the dose of Semaglutide was titrated over the first week to achieve a steady-state maintenance dose of 30 nmol/kg/day, beginning with 0.6 nmol/kg on day 1 until 30 nmol/kg was achieved on day 6. CRV431 was prepared bi-weekly at a concentration of 32 mg/mL in a self-microemulsifying drug delivery system (SMEDS), stored at 4 °C, and protected from light. The SMEDS vehicle contained 5% ethanol, 5% propylene glycol, 10% PEG 400, 15% Solutol HS15 and 65% water. The mice received daily oral gavage of CRV431 at a dose of 80 mg/kg. The 80 mg/kg dose and dosing schedule were provided by Hepion Pharmaceuticals based on prior efficacy studies with CRV431. Hepatic CRV431 concentrations were not measured, so whether co-administration with Semaglutide alters hepatic exposure of CRV431 was not determined. For both compounds, doses were adjusted based on individual body weight and measured every other day throughout the course of treatment. General health and behavior were also monitored for signs of distress and morbidity. Animals were maintained under 2.5% isoflurane anesthesia for oral gavage and subcutaneous injections. Western diet and CCl_4_ were continued through the treatment phase in all disease and drug-treated groups, whereas the regression group was returned to normal chow.

Animal experiments were conducted in compliance with the protocols approved by the Institutional Animal Care and Use Committee (IACUC) of the Scripps Research Institute (TSRI; protocol 11–0015). All staff were trained and certified per IACUC requirements. All procedures adhered to the National Institutes of Health Guide for the Care and Use of Laboratory Animals and the Code of Federal Regulations. The study design did not require implementation of humane endpoints, as animals were euthanized at predetermined experimental timepoints, at which time mice were anesthetized with 2.5% isoflurane and euthanized by cervical dislocation. The euthanasia endpoints were reviewed and approved by the IACUC. Animals in the study did not demonstrate distress criteria including significant weight loss (≥20% of body weight), emaciated appearance, lack of grooming behaviors, lethargy, dehydration, or respiratory distress, and no animals died before meeting criteria for euthanasia.

### Tissue processing and histology

At the study endpoints, mice were anesthetized with 2.5% isoflurane and euthanized by cervical dislocation. Livers were excised and fixed in buffered zinc formalin (4% formaldehyde with zinc salts; Fisher Scientific Cat# 22-050-259) for a maximum of 72 h at room temperature and then stored in 70% ethanol, followed by paraffin embedding. Formalin-fixed paraffin-embedded sections were cut at 7 µm thickness and fixed for 2 h at 60 °C.

To visualize the morphology and assess collagen deposition, liver sections were stained with hematoxylin and eosin (H&E) and picrosirius red (PSR), respectively, using established methods [16]. The stained slides were scanned with an Aperio AT2 digital pathology slide scanner (Leica Biosystems) and Aperio ImageScope. Fibrosis was quantified by calculating the percentage of Sirius Red-positive area in scanned whole-slide images using ImageJ software (Fiji V1.54; National Institutes of Health (NIH), Bethesda, MD; [17]. Histological staining and scoring, including HCC evaluation and SR quantification, were conducted in a blinded fashion using coded slides, and group identities were unblinded only after the final data processing. Individual animals were tracked by cage throughout the study. At takedown, each sample was assigned a blinded letter code, and all image-based analyses, including PSR quantification, HCC scoring, and fluorescence intensity quantification, were performed on these coded samples with the analyst blinded to treatment allocation; the letter codes were reconciled to treatment groups only after data processing was complete. Liver tumors were counted, and their diameters were measured with a ruler. Liver tumor burden (HCC) was scored on a 0–7 ordinal scale based on the number and size of macroscopic nodules (small: > 0.1–0.5 cm; medium: 0.5–1.0 cm; large: > 1 cm) [16]. Hepatic steatosis was quantified objectively as the percentage of steatotic (lipid vacuole) area on the scanned hematoxylin and eosin sections using ImageJ by segmenting white lipid vacuoles (pixels in which all three color channels were high) and retaining round, lipid-sized objects while excluding vessels and sinusoids; per section values were averaged per animal. Hematoxylin and eosin sections were available for a limited subset of animals, so steatosis is reported descriptively rather than as a group comparison. Formal NAFLD activity (NAS) and NASH-CRN scores were not assigned, as these systems were developed and validated for human liver biopsies and do not map directly onto this carbon tetrachloride accelerated murine model; disease activity was also secondary to the antifibrotic and antitumor endpoints of this study. Hepatic hydroxyproline content was measured colorimetrically (OxiSelect Hydroxyproline Assay Kit, STA-675, Cell Biolabs). Liver tissue was homogenized at 10 mg per 100 uL, acid-hydrolyzed, and assayed in duplicate with absorbance read at 550 nm against a hydroxyproline standard curve; content was expressed per mg wet tissue.

Fluorescence immunostaining was performed on the liver sections to assess inflammatory signaling and macrophage abundance. Sections were deparaffinized, rehydrated, and heat-induced antigen retrieval was performed in 10 mM sodium citrate buffer (pH 6.0) containing 0.05% Tween-20 by heating the slides to near boiling for 20 min, followed by cooling in running tap water for 10 min. The sections were washed twice with Tris-buffered saline (TBS; pH 7.6) containing 0.025% Triton X-100 (TBST) with gentle agitation. Nonspecific binding was minimized by blocking with 10% normal serum and 1% BSA and washing again with TBST. Sections were incubated with primary antibodies against Tumor Necrosis Factor alpha (TNFa; ab6671, Abcam, USA) and resident liver macrophages (F4/80; ab6640, Abcam, USA) diluted in TBS (1:1000) containing 1% BSA for one hour at room temperature, washed, and then incubated with species-appropriate fluorescent secondary antibodies (1:1000) for one hour at room temperature. Following a final TBST wash, nuclei were counterstained with Hoechst (1:2000). Images were captured using a Nikon epifluorescence microscope at 20x magnification and exported as TIFF files for downstream analysis. For alpha-smooth muscle actin, a sperate set of sections was stained by indirect immunofluorescence usinga. Rabbit polyclonal anti-ACTA2/alpha-smooth muscle actin antibody (Proteintech, 14395–1-AP; 1:1000) following the same deparaffinization, antigen retrieval, and blocking steps, detected with a Texas Red-conjugated goat anti-rabbit secondary antibody (1:500) and counterstained with Hoechst (1:2000).

Fluorescent images were captured using a Nikon epifluorescence microscope at 20x magnification, exported as TIFFs using the NIS elements viewer (Nikon Instruments; V5.22) as sixteen-bit grayscale channel TIFFs without display look-up tables applied, to preserve raw pixel intensities, and quantified using ImageJ using custom batch macros with the individual animal as the experimental unit. For each marker, background was subtracted (rolling-ball radius 50 pixels) and a single fixed intensity threshold was applied uniformly across all images in the cohort; per-image automatic thresholding was not used. Alpha-smooth muscle actin was quantified as the percentage of field area above threshold. TNF-α and F4/80 colocalization was quantified per field as Manders’ overlap coefficient, reported as the fraction of F4/80-positive area also positive for TNF-α, restricted to tissue defined by the Hoechst channel. Fields in which the 90th-percentile background intensity exceeded twice the cohort median were excluded before analysis as tissue folds or debris under a pre-specified criterion applied uniformly across animals. Per-field values were averaged to a single value per animal prior to statistical comparison. Per-field values were averaged to a single value per animal prior to statistical comparison. Hepatic steatosis was quantified on the hematoxylin and eosin sections by segmenting white lipid vacuoles (pixels all three-color channels high) and retaining round, lipid-sized objects while excluding vessels and sinusoids.

### RNA extraction and quantitative PCR

Total RNA was extracted from the liver tissue using an RNA isolation kit (Qiagen, Aarhus, Denmark) according to the manufacturer's instructions. RNA concentration and purity were assessed spectrophotometrically using a Take3 microvolume plate on a BioTek Reader. For each sample, 1.0 µg of total RNA was reverse-transcribed to first-strand cDNA using an Advantage RT-for-PCR kit (Takara Bio, Cat. no. 639505) with a mixture of random hexamers and oligo(dT) primers. The expression of housekeeping genes and hepatic stress-associated transcripts was quantified using SYBR Green-based RT-qPCR performed by CD Genomics. Primer sequences (5’-3’) were as follows: glutamic-oxaloacetic transaminase 1 (Got1; aspartate aminotransferase) forward, CAGATTGGAATGTTCAGTT; reverse, TCGGCAGGAGATAGATAT. Glutamic-pyruvic transaminase (Gpt; alanine aminotransferase) forward, CATTCCCATTCCTCAGTA; reverse, CAGGTAGTAGTCCACTTG. GAPDH served as the housekeeping gene for normalization.

### Statistics

All statistical analyses were performed using GraphPad Prism (version 10.6.0, GraphPad Software for Macintosh, Boston, Massachusetts, USA, www.graphpad.com). Normality was assessed using the Shapiro-Wilk test. For all parametric data, group comparisons were performed using one-way analysis of variance (ANOVA) with appropriate post-hoc corrections, depending on the variance structure. For data sets with homogenous variances, including body and liver weights, one-way ANOVA followed by Tukey’s multiple comparisons test was applied. When variance heterogeneity was detected, the Brown-Forsythe and Welch ANOVA were applied (picrosirius red). Because HCC burden was recorded on an ordinal 0–7 scale, it was analyzed separately by the Kruskal-Wallis test with Dunn post-hoc comparisons. All data are presented as mean ± standard error of the mean (SEM) unless otherwise stated. Statistical significance was defined as *p* < 0.05. Hepatic hydroxyproline content was compared by one-way ANOVA after confirming homogeneity of variance with the Brown-Forsythe test. Alpha-smooth muscle actin percent positive area and TNF-α/F4/80 colocalization, each summarized as one value per animal, were compared by the Kruskal-Wallis test.

For hepatic alanine aminotransferase (ALT) and aspartate aminotransferase (AST) RT-qPCR endpoints, between-group differences were evaluated by Kruskal-Wallis test with pairwise comparisons controlled using the two-stage Benjamini-Krieger-Yekutieli (BKY) false discovery rate procedure (Q = 0.05).

## Results

Body weights differed significantly across the treatment groups (Fig 1B). Regression controls exhibited gradual weight gain throughout the study, whereas vehicle- and CRV431-treated mice showed a mild weight decline but then remained constant. Groups receiving Semaglutide alone or Semaglutide in combination with CRV431 displayed pronounced and sustained reductions in total body weight. Liver weight did not correlate with body weight trends (Fig 1C). Liver weight is reported as absolute organ weight. Because mice were group housed and individual identities were not linked between the longitudinal body weight records and terminal liver collection, a per animal liver to body weight ratio could not be calculated. One-way ANOVA with multiple comparison testing indicated that liver weights in the CRV431 monotherapy group differed significantly from those in the Semaglutide (*p* < 0.0001).

**Fig 2:**
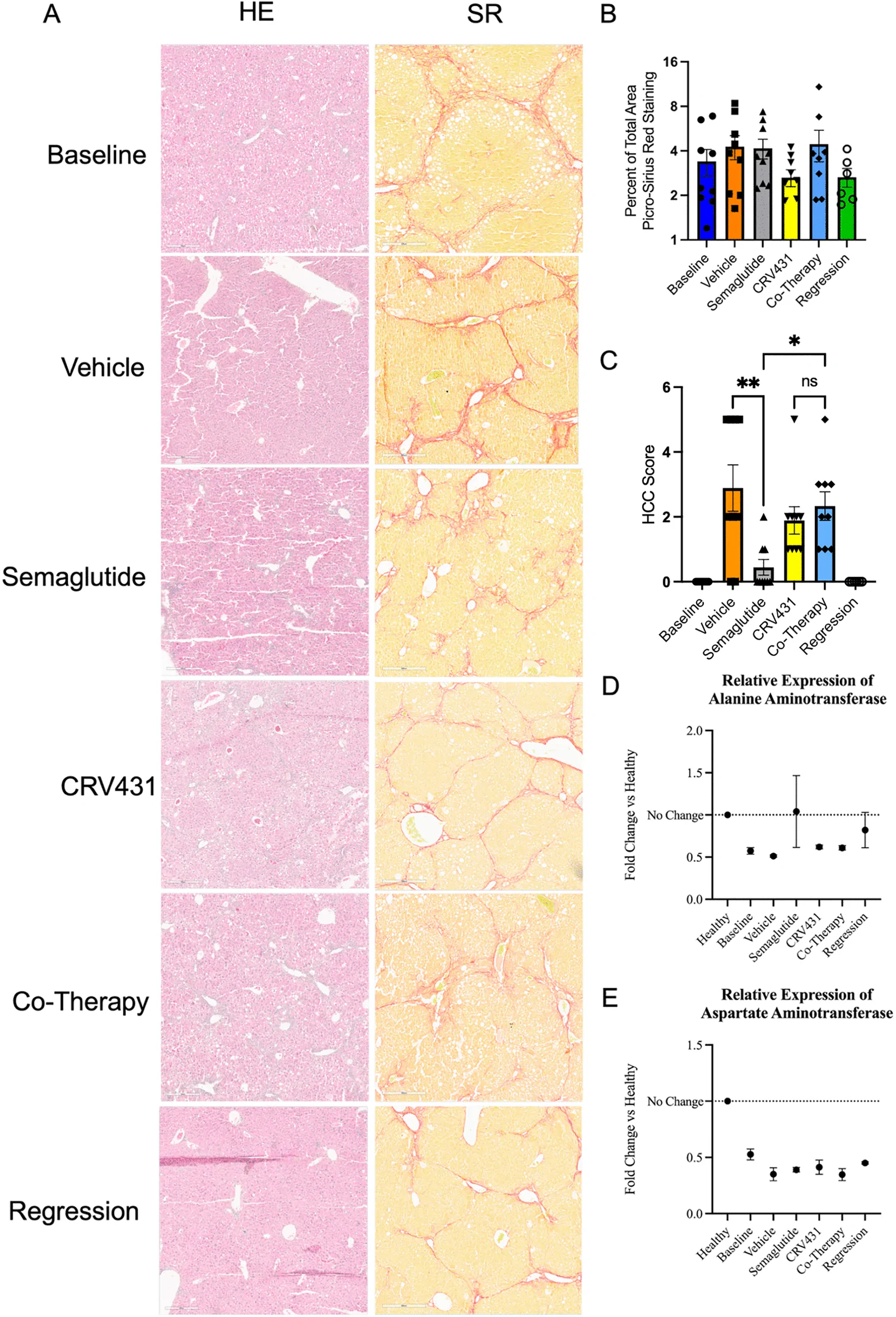
Combination therapy does not significantly alter fibrosis but modulates tumor burden. (A) Representative Picrosirius red staining of liver sections. Scale bar, 100 µm. (B) Quantification of SR-positive area (% of whole-slide area). (C) HCC burden scored on a 0-7 ordinal scale based on number and size of macroscopic nodules. (D) Relative expression of Alanine and (E) aspartate aminotransferase.

Picrosirius red staining (Fig 2A) revealed no significant differences in collagen deposition between treatment groups. Similarly, robust variance-adjusted analyses showed no overall treatment effect on SR-positive areas (Welch’s ANOVA: W(5,19.91) = 1.736, *p* = 0.1725) confirmed a lack of overall treatment effect (Fig 2B). In contrast, treatment significantly affected HCC scores (Fig 2C; Kruskal-Wallis test *H* = 32.32, *p* < 0.0001). Mean gross nodule counts were 0 in Baseline and Regression groups, and increased in Vehicle (2.89 ± 2.147 SD, n = 9), with lower values in Semaglutide (0.44 ± 0.73, n = 9) and CRV431 (1.89 ± 1.27, n = 9), and an intermediate effect in Co-therapy (2.33 ± 1.32, n = 9). Semaglutide monotherapy produced a stronger reduction in tumor burden than CRV431 monotherapy (*p* < 0.01 vs. not significant, respectively). Under these conditions, combination therapy did not demonstrate a clear enhancement beyond the monotherapy effects.

Hepatic transaminase-associated gene expression was assessed across the treatment groups and presented as ∆Ct values (target housekeeping; Fig 2D and 2E). The ALT ∆Ct values showed a broad overlap across the MASLD groups (Kruskal-Wallis, *p* = 0.149). Pairwise comparisons were performed using rank-based multiple comparisons with two-stage BKY false discovery rate correction (Q = 0.05; 21 comparisons), and no ALT comparisons met the FDR threshold (all q ≥ 0.237). Similarly, AST ∆Ct values showed a broad overlap across groups (Kruskal-Wallis, *p* = 0.136), and no pairwise differences remained significant after BKY FDR correction (all q ≥ 0.237). The log2(Got1/Gpt) transcript ratio did not differ across the groups (one-way ANOVA with Tukey’s multiple comparisons; all adjusted *p* ≥ 0.3309). Group means were negative in all experimental conditions (baseline −0.129, vehicle −0.596, Semaglutide −1.199, CRV431 −0.619, co-therapy −0.852, Regression −0.817), consistent with relatively higher Gpt than Got1 transcript abundance on average.

**Fig 3:**
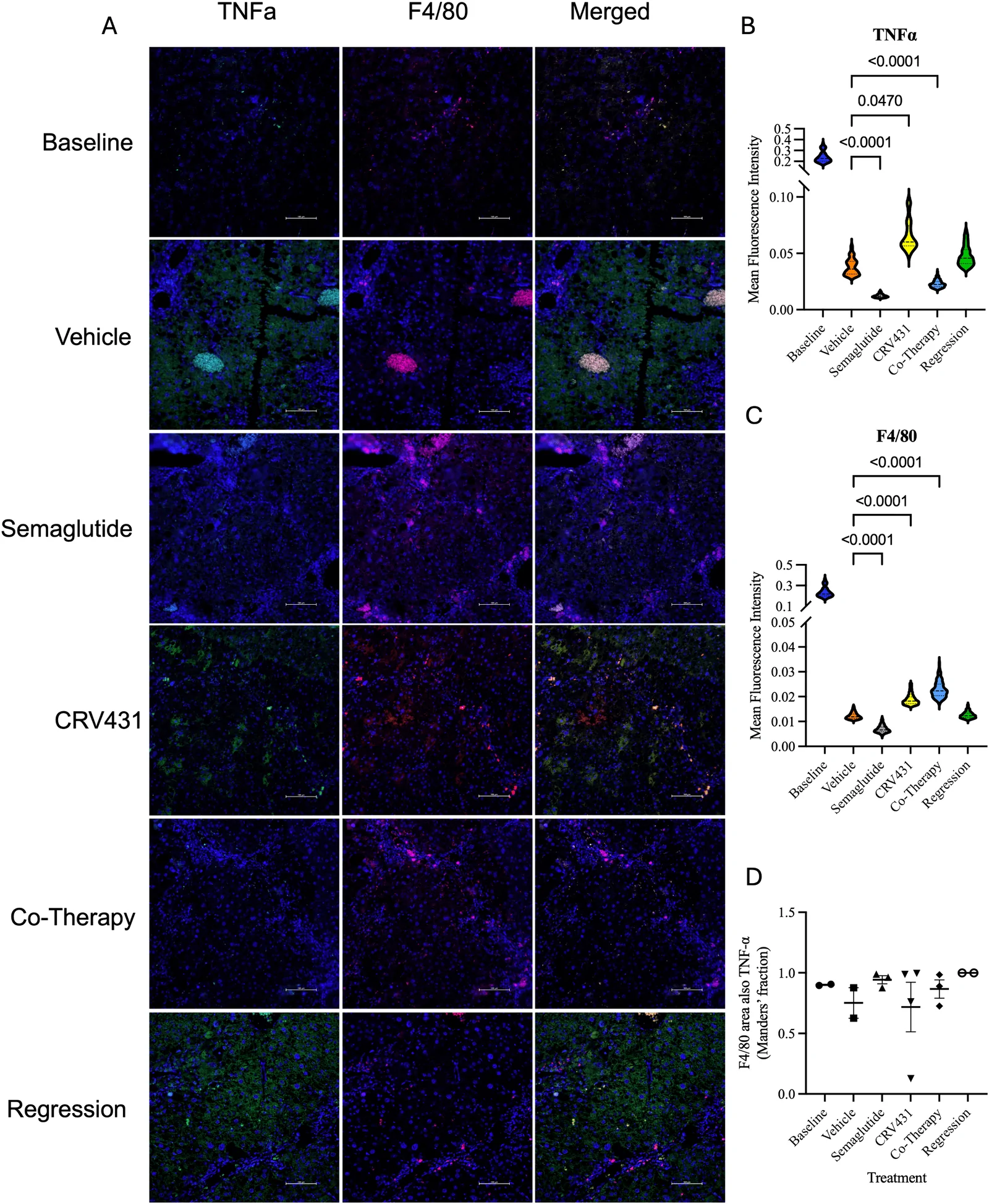
Hepatic TNF-α and F4/80 immunofluorescence. (A) Representative fluorescence images of TNF-α (Texas red), F4/80 (FITC), and Hoechst nuclear counterstain (blue). Scale bar, 100 µm. (B) Colocalization of TNF-a and F4/80 quantified per animal as the Manders’ fraction of F4/80-positive area also positive for TNF-a; each symbol is one animal and bars indicate group medians.

To determine whether the divergent metabolic and tumor responses were accompanied by changes in hepatic immune activation, we immunostained liver sections for TNA-α and F4/80 and quantified their spatial overlap at the individual-animal level using Manders’ colocalization coefficient, with the animal as the experimental unit (Fig 3A). The fraction of F4/80-positive macrophage area that was also positive for TNF-α did not differ significantly across treatment groups (Fig 3B; Kruskal-Wallis, *p* = 0.23; H = 6.86; 16 animals across six groups). At the animal level, neither monotherapy nor co-therapy significantly altered the extent of TNF-α and F4/80- colocalization, and this analysis is presented as exploratory given the small number of animals per group.

**Fig 4:**
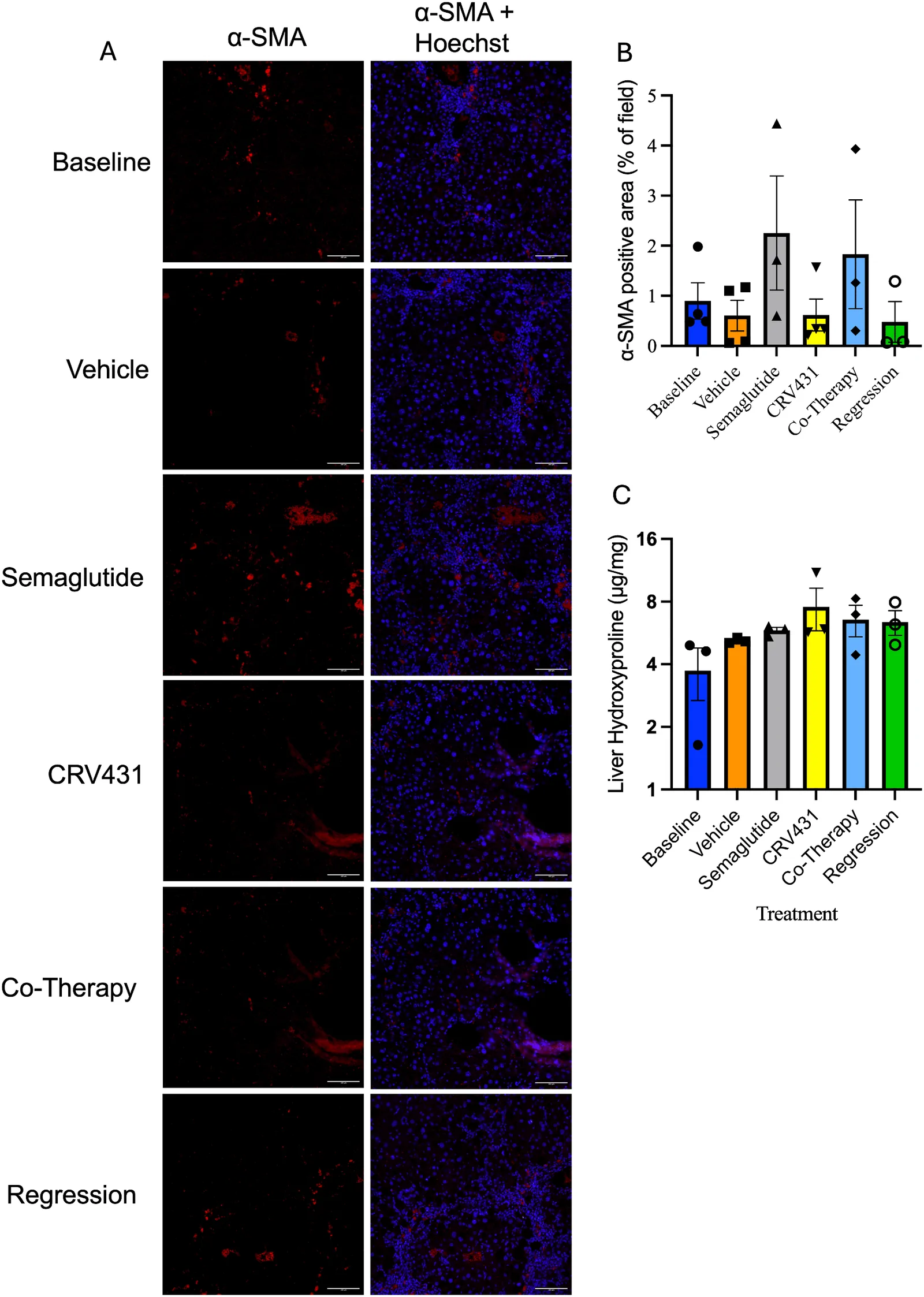
Hepatic α-smooth muscle actin and hydroxyproline across treatment groups. (A) Representative liver sections immunostained for α-smooth muscle actin (α-SMA, red) and counterstained with Hoechst (nuclei, blue), shown as the α-SMA channel alone (left) and merged with Hoechst (right), for the Baseline, Vehicle, Semaglutide, CRV431, Co-Therapy, and Regression groups (top to bottom). Images were acquired at 20x; scale bars 100 µm. (B) α-SMA positive area, quantified as the percentage of field area above a single fixed threshold, with the individual animal as the experimental unit, analyzed by Kruskal-Wallis test. (C) Liver hydroxyproline content (µg per mg wet tissue), analyzed by one-way ANOVA.

Two orthogonal fibrosis measures agreed with the picrosirius red result. Hepatic alpha-smooth muscle actin (Fig 4A), a marker of hepatic stellate cell activation, was quantified as percent positive area and showed no significant difference across groups (Fig 4B; Kruskal-Wallis, *p* = 0.33; 21 animals). Hepatic hydroxyproline content, an orthogonal biochemical measure of total collagen, likewise did not differ significantly across groups (Fig 4C; µg per mg wet tissue, mean ± SEM: Baseline 0.50 ± 0.14, Vehicle 0.69 ± 0.01, Semaglutide 0.78 ± 0.02, CRV431 1.01 ± 0.23, Co-therapy 0.87 ± 0.15, Regression 0.85 ± 0.12; one-way ANOVA F(5,12) = 1.657, *p* = 0.2192, with homogenous variances confirmed by the Brown-Forsythe test, *p* = 0.7374; n = 3 per group), and no treatment arm fell below vehicle, concordant with the picrosirius red result. Hepatic steatosis, assessed on available hematoxylin and eosin sections, was mild overall (grade 0–1; approximately 2–8% steatotic area) and is reported descriptively.

## Discussion

This study evaluated the effects of Semaglutide, CRV431, and their co-administration in a diet- and CCl_4_-driven MASLD model exhibiting advanced liver injury. Hepatic Got1 and Gpt transcript abundance, a molecular readout distinct from serum enzyme activity, was not markedly elevated at the endpoint [18]. We did not measure serum transaminase activity in this study, and we note this as a limitation; the transcript data are not interpreted as circulating enzyme levels. Overall, the data indicate that Semaglutide and CRV431 exert divergent biological effects, and that the combined treatment does not yield additive benefits across core histologic and molecular endpoints. As expected, Semaglutide induced pronounced and sustained reductions in body weight, which was consistent with its established metabolic actions. In contrast, CRV431 monotherapy did not significantly alter the total body mass, further supporting its characterization as an antifibrotic and hepatoprotective molecule rather than a metabolic modulator. Notably, liver weight did not scale with total body weight across groups, reinforcing the notion that hepatic remodeling in this model is uncoupled from overall adiposity and is more tightly linked to localized inflammatory and regenerative processes. Because individual animals were not tracked between the longitudinal body weight records and terminal liver collection, liver weight is reported as an absolute organ weight and is not adjusted for body size, which is a limitation when comparing groups that differed in body weight, such as the Semaglutide treated arms.

Despite substantial metabolic effects, fibrosis severity was not detected with any single or combined treatment. Picrosirius red quantification showed no significant treatment effect across the groups, and co-therapy did not outperform CRV431 monotherapy. The two orthogonal fibrosis measures added for this study agreed with this result: hepatic hydroxyproline content, a biochemical index of total collagen, and alpha-smooth muscle actin, a marker of stellate cell activation, likewise did not differ significantly across groups, so three independent readouts concordantly indicate no antifibrotic benefit in this cohort. Although the CRV431 group exhibited a modest numerical decrease in the Sirius Red-positive area, this trend was not statistically significant in the present cohort. While this directionality is consistent with prior reports of CRV431-mediated reductions in collagen deposition, the effect size may have been constrained by the severity of the model: prolonged Western diet exposure with repeated CCl_4_ hepatotoxicity can drive persistent extracellular matrix deposition and limit measurable regression within an 8-week therapeutic window [19,20]. This study also has limitations of statistical power. No a priori power calculation was performed, and group sizes were modest and were further reduced in some arms by attrition during the study. As several of the primary findings are negative, we cannot fully distinguish a true absence of effect from an underpowered comparison, and we report the effect size and a posteriori power for the fibrosis endpoint (Cohen d and eta squared with confidence intervals) to make this explicit. For the picrosirius red endpoint the omnibus effect size was modest (eta squared = 0.12, 90% CI 0.00 to 0.20; Cohen f = 0.38), and the a posteriori power to detect an effect of this size at alpha = 0.05 with the present group sizes was only 0.46, confirming the fibrosis comparison was underpowered. The CRV431 versus vehicle contrast, the direction noted above, corresponded to a Cohen d of 0.89, a large, standardized difference that did not reach significance in this cohort.

In contrast to fibrosis, HCC burden was strongly improved by Semaglutide, consistent with previous studies showing that GLP-1 receptor agonism reduces hepatic steatosis and improves metabolic parameters in both clinical and preclinical settings [21–23]. Semaglutide reduced the tumor burden without a corresponding shift in SR, suggesting that antitumor activity in this model may be mediated through mechanisms partially uncoupled from bulk collagen content, such as changes in systemic metabolic load, hepatocellular stress, or the regenerative microenvironment.

Fluorescence profiling did not reveal a treatment effect on hepatic macrophage inflammation. TNF-α and F4/80 colocalization, quantified per animal, did not differ significantly across groups, indicating that neither Semaglutide nor CRV431, alone or in combination, measurably altered the extend of TNF-α-macrophage association in this cohort. This null result is limited by the small number of animals per group and is presented as exploratory.

These results suggest that, in advanced MASLD with established fibrosis and emergent HCC, GLP-1-mediated metabolic remodeling and cyclophilin inhibition do not converge into an additive or synergistic therapeutic response. Several factors may explain this discrepancy. First, the lack of synergy in our co-treatment group may be due to the distinct biological and stage-dependent constraints that each compound modulates. GLP-1 receptor agonists engage in both central and peripheral mechanisms, including a reduction in food intake via hypothalamic pathways and direct modulation of hepatocellular metabolism. Semaglutide has been shown to reduce *de novo* lipogenesis by downregulating SREBP-1c and ChREBP, enhance β-oxidation via AMPK activation, and improve mitochondrial function through increased PGC-1a expression [3,24,25]. These effects plausibly reduce steatosis and oxidative stress, which are thought to indirectly influence extracellular matrix remodeling but may not directly suppress the transcriptional programs driving myofibroblast activation or dismantle established extracellular matrices over a short therapeutic window, consistent with the minimal change in picrosirius red fibrosis observed in this cohort.

CRV431, in contrast, acts downstream of hepatocyte lipotoxicity by targeting cyclophilin isoforms that regulate multiple arms of the fibrogenic and HCC cascade. Cyclophilin A (CypA) promotes TGF-b/SMAD signaling [26], while Cyclophilin B (CypB) facilitates procollagen maturation in the endoplasmic reticulum via its role as a chaperone [11]. In contrast, cyclophilin D (CypD) is a mitochondrial cyclophilin that regulates the opening of the mitochondrial permeability transition pore, thereby shaping mitochondrial stress responses and cell death susceptibility in the context of liver injury [27,28]. Inhibition of cyclophilin isoforms disrupts collagen deposition, reduces hepatic stellate cell activation, and attenuates pro-inflammatory cytokine release [6,9,10,29]. Therefore, CRV431 acts independently of metabolic flux, making it particularly appealing as a fibrosis- and HCC-specific therapeutic agent. However, when combined with Semaglutide, it failed to amplify antifibrotic outcomes. One possibility is that Semaglutide-induced alterations in hepatic lipid content, redox balance, and bile acid metabolism modulate the intracellular distribution or stability of CRV431, diminishing its ability to accumulate in fibrogenic niches [30,31]. Alternatively, the activation state of stellate cells in the setting of chronic CCl_4_ injury may be refractory to cyclophilin inhibition if upstream inflammatory or wound-healing signals remain active despite metabolic correction and if fibrotic architecture is entrenched, limiting measurable regression during the treatment interval.

Another consideration is the potential antagonism between Semaglutide’s metabolic remodeling and CRV431’s inhibition of peptidyl-prolyl isomerases. Cyclophilins have pleiotropic roles beyond fibrosis, including mitochondrial protein folding, NF-kB signaling, and unfolded protein response modulation. We surmise that GLP-1-induced shifts in endoplasmic reticulum stress or mitochondrial dynamics could reduce CRV431 efficacy; however, our study did not directly test these mechanisms, and they should be interpreted as hypotheses motivating future studies [32]. A parsimonious explanation is that treatment was initiated after fibrotic architecture and tumorigenic processes were already established, leaving a limited capacity for short-interval regression in collagen burden. In this context, CRV431’s antifibrotic mechanism may be constrained by the durability for extracellular matrix scaffolding and the persistence of injury-driven wound healing cues. Fibrosis regression is an active remodeling process that requires scar-associated macrophages and collagenolytic programs, including macrophage-associated MMP13. We postulate Semaglutide-mediated suppression of hepatic inflammation could paradoxically blunt the recruitment or activation of matrix-remodeling macrophage populations needed to clear pre-existing scars [33]. Conversely, the effects of Semaglutide on mouse weight and tumor burden indicate that metabolic modulation can retain efficacy even at advanced disease stages, but does not necessarily translate to measurable collagen regression on the timescale studied [18].

## Conclusion

In summary, Semaglutide and CRV431 engage distinct biological pathways that do not yield additive therapeutic benefits when administered once MASLD progresses to established fibrosis and early HCC. Semaglutide treatment demonstrated potent metabolic and antitumor effects, whereas CRV431 produced only modest reductions in collagen deposition, as assessed by PSR, and did not measurably reduce fibrosis over the treatment interval. These effects did not improve with co-therapy when delivered in combination, indicating that cyclophilin inhibition and GLP-1-mediated metabolic remodeling did not interact in a way that enhanced hepatic repair once injury was incurred and advanced. At the animal level, TNF-α and F4/80 colocalization did not differ significantly across groups, and this exploratory inflammatory readout showed no benefit of co-therapy over monotherapy. Hepatic ALT and AST transcript measurements overlapped across groups and did not yield FDR-significant pairwise separation, consistent with the limited treatment-associated shifts in these transaminase-associated mRNA endpoints.

Our previous findings indicated that CRV431 showed the greatest efficacy when cyclophilin activity was responsive to modulation, and dosing was administered prophylactically, preceding the onset of hepatic injury [6]. The present results underscore that the therapeutic window for cyclophilin-directed action may lie earlier in disease evolution, whereas GLP-1 receptor agonists retain efficacy in advanced MASLD. This distinction has important translational implications in patient stratification and treatment sequencing. Therefore, further research is needed to clarify the mechanistic constraints limiting the lack of synergy between metabolic modulation and cyclophilin inhibition in MASLD and determine whether earlier intervention or modified dosing paradigms can realign these otherwise complementary pathways towards additive benefits. Future studies should incorporate earlier intervention time points, longer treatment windows, and direct stellate cell activation and matrix remodeling readouts to define when cyclophilin inhibition can contribute meaningfully to combination regimens.

## Acknowldgements

The authors gratefully acknowledge the Scripps Histology Core for tissue-embedding services and the Scripps Research Vivarium staff for expert animal care, including cage maintenance and routine health monitoring.
