# Supplementary material for: Divergent effects of semaglutide and CRV431 Co-therapy on liver fibrosis and HCC in MASLD mouse model
Source: PLoS One. 2026 Sep 18;21(9):e0358225. doi: 10.1371/journal.pone.0358225 (PMC13588371; doi:10.1371/journal.pone.0358225)
Supplement: Supplementary file 2 [file pone.0358225.s003.docx]

Response to Reviewers

Manuscript: PONE-D-26-13636

Title: Divergent Effects of Semaglutide and CRV431 Co-Therapy on Liver Fibrosis and HCC in a MASLD Mouse Model

Journal: PLOS ONE

Dear Reviewers,

Thank you for the careful and constructive evaluation of our manuscript. The reviewers raised a consistent and well-founded set of concerns, and we have revised the manuscript substantially in response. The most important additions are orthogonal fibrosis and disease activity measures (hydroxyproline, and alpha-SMA immunostaining)**,** a corrected and internally consistent statistical framework in which ordinal HCC scores are analyzed by Kruskal-Wallis and continuous fibrosis data by Welch ANOVA, and a study design schematic with explicit group definitions and timeline.

Below we respond to each point in turn. Reviewer and editor comments are reproduced in italics, followed by our response. New or revised text in the manuscript is indicated by the relevant section.

Where a comment recommends new experiments, the response reflects analyses and assays we are completing for the revision. A small number of figure level corrections, for example the axis label change from Both to Co-therapy, are made directly in the figure files.

Editor and Journal Requirements

1. Style and file naming requirements.

Response: We have reformatted the manuscript to follow the PLOS ONE main body and title, author, and affiliation templates, including file naming, section order, figure callouts, and reference style.

2. Code sharing.

Response: No bespoke analysis code underpins the findings. All quantification was performed in ImageJ or Fiji and GraphPad Prism using standard built in routines. The ImageJ macros used for picrosirius red area fraction, fluorescence intensity normalization, and Coloc2 colocalization have been deposited alongside the data in the Dryad record and are available without restriction.

3. Grant information mismatch between Funding Information and Financial Disclosure.

Response: We have reconciled the two fields. The single supporting award is National Institutes of Health grant 5R01AI143931-05 to Philippe Gallay. The corrected statement is provided in the cover letter.

4. Role of funder statement.

Response: The funder had no role in study design, data collection and analysis, decision to publish, or preparation of the manuscript. We have included this statement in the cover letter for the submission form. Hepion Pharmaceuticals supplied CRV431 but had no role beyond provision of the compound, as stated in the Conflicts of Interest Disclosure.

5. Expansion of NIH acronym.

Response: The funder is now stated in full as the National Institutes of Health (NIH) in the Financial Disclosure and cover letter.

6. Data availability.

Response: All underlying data are deposited in Dryad (doi:10.5061/dryad.2fqz6134j), including per animal values behind every mean and variance measure for body and liver weight, picrosirius red area fraction, hydroxyproline, HCC scores, fluorescence intensities, colocalization, and qPCR. The repository will be released publicly on acceptance. We have updated the Data Availability Statement to reflect unrestricted public access on publication.

7. Ethics statement placement.

Response: The standalone Ethics Statement that previously appeared after the Data Availability Statement has been removed. The IACUC approval and NIH guideline compliance language now appears only within the Methods, in the MASLD Induction and Experimental Design subsection under protocol 11-0015.

8. Reviewer recommended citations.

Response: We have evaluated the literature referenced by the reviewers and incorporated the relevant citations where they strengthen the disease model rationale and the antifibrotic comparison, as noted in the responses below.

Reviewer 1

We thank Reviewer 1 (Dr. Maria Dermit) for recognizing the translational value of the stage dependent therapeutic window interpretation and for a rigorous methodological critique. We address each concern below.

Major concern 1. Underpowered primary endpoint.

*No a priori power calculation, effect size, or confidence intervals are reported for the picrosirius red quantification. With n equal to 9 per group, the study may be underpowered to detect meaningful differences in collagen deposition.*

Response: We agree and have added both an a posteriori power and effect size assessment for the picrosirius red endpoint. For the omnibus comparison we now report the effect size as eta squared with its 90 percent confidence interval (eta squared = 0.12, 90% CI 0.00 to 0.20; Cohen f = 0.38), and for the key contrast of CRV431 versus vehicle we report Cohen d (d = 0.89). Given the observed within group variance, the cohort of n equal to 9 per group provided a posteriori power of 0.46 to detect an effect of the observed size at alpha = 0.05. We have added a sentence to the Statistics subsection describing how n was chosen and a paragraph to the Discussion that explicitly distinguishes a true null result from an underpowered comparison, and we now frame the fibrosis finding as no detectable difference under the present power rather than as proof of no effect.

Major concern 2. Fibrosis quantification insufficient for the primary claim.

*PSR area fraction is the sole fibrosis readout. The authors should add alpha-SMA immunostaining, hydroxyproline content, and semiquantitative fibrosis staging.*

Response: We have strengthened the fibrosis assessment with two orthogonal measures. We now report hepatic hydroxyproline content as a biochemical measure of total collagen and alpha-smooth muscle actin immunofluorescence as an index of hepatic stellate cell activation, providing three independent fibrosis readouts together with picrosirius red. Neither measure differed significantly across groups, and no treated arm fell below vehicle, concordant with the picrosirius red result and supporting the conclusion that no antifibrotic benefit was detected; the values and statistics are reported in the Results and figures. We did not include formal NASH-CRN fibrosis staging, because reliable staging requires a qualified pathologist reading coded slides, which was not available for this study; the biochemical (hydroxyproline) and image-based (picrosirius red, alpha-SMA) measures provide the orthogonal quantitative assessment the reviewer requested.

Major concern 3. Absence of MASH activity scoring.

*No NAS or MASH-CRN scoring is reported for steatosis, lobular inflammation, or hepatocyte ballooning.*

Response: We appreciate this suggestion but did not assign formal NAS or MASH-CRN activity scores. Reliable NAS and MASH-CRN scoring requires a qualified pathologist reading coded slides, which was not available for this study. In addition, these systems were developed and validated for human liver biopsies, and their application to a carbon tetrachloride accelerated murine model, in which hepatotoxin driven pericentral injury does not correspond directly to the human rubric, has recognized limitations, and disease activity was a secondary consideration relative to our antifibrotic and antitumor endpoints. To address the steatosis component objectively, we instead quantify hepatic steatosis as the percentage of steatotic area on the scanned H&E sections using automated image analysis, and hepatic inflammation is quantified by TNF-alpha and F4/80 immunofluorescence. We have added this rationale to the Methods and note the absence of formal activity scoring as a limitation.

Major concern 4. Colocalization endpoint analytically invalid as presented.

*TNF-alpha and F4/80 colocalization values were summarized at the group level without mouse level replicates. The panel should be re-quantified at the individual animal level with statistical testing or removed.*

Response: We agree, and we have re-quantified this endpoint at the individual-animal level as the reviewer requested rather than removing it. We re-stained and imaged additional sections so that each group is represented by multiple animals (16 animals across six groups), quantified TNF-alpha and F4/80 colocalization for every field in ImageJ using Manders' overlap coefficient, averaged fields to a single value per animal, and treated the animal as the experimental unit for all statistics. The fraction of F4/80-positive macrophage area also positive for TNF-alpha did not differ significantly across treatment groups (Kruskal-Wallis, p = 0.23; 16 animals). The former group-level colocalization summary (former Figure 3D) and the per-segmented-cell intensity bar graphs, which were not valid for inference, have been removed and replaced by this animal-level analysis. The Results, Methods, and figure legend have been revised accordingly. Because the omnibus test was not significant, no pairwise comparisons are reported, and given the small number of animals per group we present this endpoint as exploratory.

Major concern 5. Transaminase endpoints are non-standard.

*ALT and AST are measured as hepatic mRNA rather than serum enzyme activity. If serum was collected these should be replaced with conventional activity assays.*

Response: Serum was not collected in this study, so serum ALT and AST enzyme activity could not be measured; any archived blood would also be unsuitable for transaminase activity after prolonged storage. We therefore do not report a serum assay. To remove the conflation the reviewer identified, the hepatic Got1 and Gpt data are now labeled throughout as transcript abundance (mRNA), not enzyme activity; the Discussion no longer draws circulating enzyme or injury conclusions from them; the AST to ALT ratio has been removed; and we state the absence of serum transaminases explicitly as a limitation.

Major concern 6. Sex as a biological variable.

*The study uses male mice exclusively with no stated justification. Either inclusion of female animals or explicit scientific justification is required.*

Response: We have added an explicit scientific justification for the male only design in the Methods and acknowledged it as a limitation in the Discussion. The Friedman two hit model used here was characterized in male C57BL/6J mice, in which the carbon tetrachloride driven fibrosis and HCC phenotype is more uniform and penetrant; females show slower and more variable progression in this model, which would have confounded the stage matched comparison central to our design. We note that sex differences in MASLD progression and GLP-1 pharmacology are precisely why a combined cohort would require its own powered design, and we now state that extension to female animals is an important direction for follow up work. If the editor considers a female cohort essential for this submission rather than a future study, we would welcome guidance, as generating a stage matched female cohort would require a new induction timeline beyond the present revision window.

Minor concern 7. Abstract statistical inconsistency.

*The abstract states HCC burden was assessed by Brown-Forsythe ANOVA, whereas the text reports Kruskal-Wallis. The correct test must be stated consistently.*

Response: We have corrected this throughout, and the correction also addresses Reviewer 3. Because HCC score is an ordinal 0 to 7 measure, it is now analyzed by Kruskal-Wallis with Dunn post hoc comparisons in the Methods, Results, and abstract (H = 32.32, p < 0.0001). The continuous picrosirius red endpoint is reported consistently by Welch ANOVA (W(5, 19.91) equal to 1.736, p equal to 0.1725). The redundant Brown-Forsythe line for the fibrosis endpoint has been removed so that one test is reported per endpoint.

Minor concern 8. No pharmacological rationale for CRV431 dosing in co-therapy.

*The combination arm uses the same 80 mg/kg oral CRV431 dose as monotherapy with no PK justification.*

Response: We have added a short rationale to the Methods and Discussion. The 80 mg/kg oral dose was selected to match the exposure used in prior single agent CRV431 efficacy studies in murine fibrosis, so that the co-therapy arm tests the addition of GLP-1 agonism against an established effective cyclophilin inhibitor dose rather than a re-optimized one. We did not measure hepatic CRV431 concentrations in the co-therapy arm, and we now state this as a limitation, noting that Semaglutide induced changes in hepatic lipid and bile acid handling could in principle alter CRV431 distribution. Direct hepatic exposure verification in the combination setting is identified as a needed follow up.

Minor concern 9. Figure label inconsistency (Both versus Co-therapy).

*The HCC score x-axis in Figure 2C labels the combination group as Both, while all other figures and the text use Co-therapy.*

Response: Corrected. The Figure 2C axis label has been changed from Both to Co-therapy so that the group name is identical across all figures and text.

Minor concern 10. Discussion mechanistic claims are speculative without supporting data.

*The hypotheses regarding bile acid mediated changes in CRV431 accumulation and GLP-1 mediated macrophage suppression are interesting but unsupported. Consider whether existing data such as MMP13 staining could anchor at least one proposal.*

Response: We have revised the Discussion to clearly delineate hypothesis from interpretation, using explicit signposting so that data supported statements and mechanistic speculation are not conflated. We did not ad MMP13 immunostaining. Within the timeframe of this revision we could not perform additional immunostaining, and MMP13 is not required to support the conclusion, which rest on the fibrosis, HCC, and existing inflammatory readouts. Accordingly, the scar resolution proposal and other mechanistic ideas are now presented explicitly as hypotheses that motivate future work, rather than as claims supported by data in this study, so no additional staining is needed to keep the Discussion appropriately cautious.

Reviewer 2

We thank Reviewer 2 for noting the clinical relevance and the innovation of pairing a GLP-1 receptor agonist with a cyclophilin inhibitor. We address the general, methods, and results points in order.

General points

General 1. Group descriptions lack clarity.

Response: We have added a clear description of all six groups (Baseline, Vehicle, Semaglutide, CRV431, Co-therapy, and Regression), stating the condition each represents and when it was assessed, and a new study design schematic (Figure 1A) showing group allocation and timeline. The text now specifies explicitly which comparisons are intended, namely each monotherapy and co-therapy against vehicle, and co-therapy against each monotherapy.

General 2. Regression group results are not discussed.

Response: We have expanded the Results and Discussion to interpret the Regression arm, which models the effect of dietary withdrawal alone. The Regression data are now reported across body and liver weight, fibrosis, HCC, transaminases, and inflammatory markers, and are discussed as the reference for the reversible component of injury, which provides context for the limited collagen regression seen with pharmacologic treatment over the eight week window.

Methods points

Methods 1. Diet source.

Response: The Western diet was purchased commercially. We have added the supplier and catalog number TD.120528, Inotiv Inc. Madison, WI, USA, along with the 42% kcal from fat specification and the 10% weight per volume sucrose in drinking water.

Methods 2. Induction duration discrepancy.

Response: Thank you for catching this. We have reconciled the timeline so that the Methods and the Figure 1 schematic state the same induction duration of 10 weeks. The figure axis and the Methods now use identical units and reference points for the 10-week induction and the six-week treatment phase.

Methods 3. CCl4 injection duration.

Response: We have specified that twice weekly intraperitoneal CCl4 was administered throughout the induction period and clarified it continued during the treatment phase, with the exact start and stop points now shown on the Figure 1A schematic.

Methods 4. Regression group implementation.

Response: We have described how dietary withdrawal was implemented for the Regression arm, namely discontinuation of the Western diet and sucrose water with return to standard chow and plain water at the start of the treatment phase, with CCl4 handling stated explicitly, while no drug or vehicle gavage was given.

Methods 5. BSA in Semaglutide storage.

Response: Clarified. BSA at 0.1 percent was included to minimize adsorption of the peptide to labware and storage surfaces, not for an *in vivo* purpose. The wording has been corrected from absorption to adsorption.

Methods 6. Subcutaneous compound not specified.

Response: We have specified that Semaglutide was the compound administered subcutaneously, whereas CRV431 and the vehicle were administered by oral gavage. The Methods now name the route for each compound.

Results points

Results 1. Figure 1 labeling, normalization, and group composition.

Response: We have relabeled Figure 1 panels as A and B and C and applied consistent panel labeling throughout. Liver weights are reported as absolute organ weights. Because animals were group housed and individual identities were not linked between the longitudinal body weight records and the terminal liver collection, we are unable to calculate a per-animal liver to body weight ratio for this cohort, and we now state this in the Results and Methods and note it as a limitation in the Discussion. Mean terminal body weights are shown in Fig1B for context, and we note that the Semaglutide and co-therapy groups had lower body weights, which we consider when interpreting the absolute liver weights. The text now states explicitly which groups remained on the Friedman protocol and which returned to standard chow (Regression).

Results 2. Terminal fasting glycemia.

Response: We do not report terminal blood glucose. Glucose was measured on whole blood that had been stored at -80˚C for over a year and read on a point of care glucometer, a platform validated only for fresh whole blood. Freeze and thaw hemolysis, together with the non-fasted and isoflurane exposed state at collection, make these values unreliable as a measure of glycemic control, so we have omitted the results. Fasting glycemia was no obtained in this study, which we now note as a limitation.

Results 3. Steatosis in more than one group, model fidelity, and neutral lipid quantification.

Response: Oil Red O neutral lipid staining requires frozen liver, which was not banked for this cohort, so this specific stain cannot be performed. Hepatic steatosis is instead assessed objectively from the scanned hematoxylin and eosin sections by automated image analysis (percent steatotic area), and we discuss the steatosis pattern across groups in the context of the two-hit Western diet plus CCl4 (Friedman) model, including where it recapitulates and where it diverges from human MASLD histology. Disease activity was a secondary consideration relative to the antifibrotic and antitumor endpoints of this study.

Results 4. Serum ALT and AST.

Response: As described in our response to Reviewer 1 major concern 5, serum was not collected, so serum ALT and AST activity could not be measured. The hepatic transaminase data are retained and clearly labeled as transcript abundance (mRNA), and the absence of serum transaminases is noted as a limitation.

Results 5. Figure 2C Both versus Co-therapy.

Response: Corrected to Co-therapy, consistent with Reviewer 1 minor concern 9.

Results 6. Col1a1 and Col3a1 transcripts.

Response: We did not add Col1a1 and Col3a1 transcript profiling. Fibrosis is the primary endpoint of this study and did not differ across groups by picrosirius red area fraction or by the orthogonal collagen measures we have added (hydroxyproline and alpha-SMA). Adding Col1a1 and Col3a1 transcripts would require new RNA extraction and qRT-PCR and, given the concordant negative fibrosis result, would not change the interpretation. We therefore did not pursue these transcripts.

Results 7. Figure 3 panel A merged columns versus panel D.

Response: As described in our response to Reviewer 1 major concern 4, the former panel D (group-level colocalization) and the per-segmented-cell TNF-alpha and F4/80 bar graphs have been replaced by an animal-level colocalization analysis (Manders' coefficient; 16 animals; Kruskal-Wallis, p = 0.23). Figure 3 now shows the representative images together with the per-animal colocalization quantification, so the discrepancy the reviewer noted between the merged panel A image and the former panel D no longer applies.

Reviewer 3

We thank Reviewer 3 for the constructive assessment and for highlighting the regression arm as a strength. We address the major and minor comments below.

Major comments

Major 1. Six groups never clearly explained; add group descriptions and a flow diagram.

Response: We have added explicit definitions for all six groups in the Methods and a new study design schematic (Figure 1A) showing the groups, induction, treatment duration, and endpoint, which also addresses the corresponding requests from Reviewer 2.

Major 2. Sample size and power.

Response: We have added the basis for the sample size and an a posteriori power and effect size assessment for the primary fibrosis endpoint, and we explicitly acknowledge in the Discussion that several conclusions are negative findings and distinguish a true null from an underpowered comparison. This is detailed in the response to Reviewer 1 major concern 1.

Major 3. Blinding across analyses.

Response: We have clarified blinding throughout the Methods. HCC evaluation and picrosirius red quantification used coded slides unblinded only after final data processing. We now state explicitly which additional analyses (alpha-SMA immunostaining and fluorescence intensity quantification) were performed blinded, and we note any endpoint where blinding was not applicable, such as automated intensity thresholding performed identically across coded images.

Major 4. Pre-treatment fibrosis and steatosis severity.

Response: The Baseline group, assessed at the start of the treatment phase, provides a cross-sectional reference for disease severity before pharmacologic intervention, and we now make this role explicit and report Baseline fibrosis, steatosis, and HCC alongside the treated groups. We did not perform longitudinal noninvasive imaging within animals, and we now acknowledge in the limitations that paired noninvasive imaging, for example magnetic resonance based measures, would allow within animal change to be quantified and is a valuable design for future studies.

Major 5. Abstract versus Methods and Results statistics.

Response: Resolved as described for Reviewer 1 minor concern 7. The Methods and Results now name the specific test for each endpoint, matching the abstract, namely Welch ANOVA for the continuous fibrosis endpoint and Kruskal-Wallis for ordinal HCC.

Major 6. HCC ordinal data analyzed with an ANOVA type test.

Response: We agree. HCC scores are now analyzed by Kruskal-Wallis with Dunn post hoc comparisons rather than a variance adjusted ANOVA, which is appropriate for ordinal data and consistent with the abstract.

Major 7. mRNA versus serum transaminase wording in the Discussion.

Response: We have corrected the wording. Because serum was not available, we do not report serum transaminase activity; the Got1 and Gpt data are now described only as hepatic transcript abundance (mRNA), and the Discussion no longer equates transcript abundance with circulating enzyme activity. The references to an AST to ALT ratio and to the sensitivity of circulating transaminases have been removed, and we note the absence of serum transaminases as a limitation.

Minor comments

Minor 1. Animal identification and tracking.

Response: We have added that animals were tracked by cage throughout induction, treatment, and endpoint.

Minor 2. Workflow flow diagram.

Response: Added as the new Figure 1 schematic covering induction, group allocation, treatment duration, and endpoint timing for each arm.

Minor 3. Multi line graph readability.

Response: We have revised the line graphs to use a colorblind friendly palette together with differentiated line styles and markers so that groups are distinguishable in color and in grayscale.

Minor 4. Baseline group not identifiable in the body weight panel.

Response: Clarified in the Figure 1 legend. The Baseline group was assessed throughout the diet induction period and subsequently euthanized prior to treatment. It therefore appears in the liver weight bar chart, and in the early timepoints of the body weight panel, but not as a longitudinal line across the treatment period.

Minor 5. Previous findings citation.

Response: We have added the specific citation or citations for our prior CRV431 work referenced in the Conclusion regarding cyclophilin activity responsiveness and prophylactic dosing: Kuo J, Bobardt M, Chatterji U, Mayo PR, Trepanier DJ, Foster RT, et al. A Pan-Cyclophilin Inhibitor, CRV431, Decreases Fibrosis and Tumor Development in Chronic Liver Disease Models. J Pharmacol Exp Ther. 2019 Nov;371(2):231–41. doi:10.1124/jpet.119.261099.

Minor 6. Peer review history option.

Response: Noted. We will indicate our preference for publication of the peer review history on the submission form.

We are grateful for the reviewers time and believe the manuscript is considerably strengthened by these revisions. We hope the revised version is now suitable for publication in PLOS ONE and welcome any further guidance.

Sincerely,

Asha Z. Goodman, Ph.D.

On behalf of all co-authors

Department of Immunology and Microbiology, Scripps Research, La Jolla, California
