# Supplementary material for: Divergent effects of semaglutide and CRV431 Co-therapy on liver fibrosis and HCC in MASLD mouse model
Source: PLoS One. 2026 Sep 18;21(9):e0358225. doi: 10.1371/journal.pone.0358225 (PMC13588371; doi:10.1371/journal.pone.0358225)
Supplement: Peer review history

# Peer review: Decision Letter 0

**DOI:** 10.1371/journal.pone.0358225.r001

**Authors:** Farhadul Islam

16 Jun 2026

Dear Dr. Goodman,

Thank you for submitting your manuscript to PLOS ONE. After careful consideration, we feel that it has merit but does not fully meet PLOS ONE’s publication criteria as it currently stands. Therefore, we invite you to submit a revised version of the manuscript that addresses the points raised during the review process.

Please submit your revised manuscript by Jul 31 2026 11:59PM. If you will need more time than this to complete your revisions, please reply to this message or contact the journal office at plosone@plos.org. When you're ready to submit your revision, log on to https://www.editorialmanager.com/pone/ and select the 'Submissions Needing Revision' folder to locate your manuscript file.

- A letter that responds to each point raised by the academic editor and reviewer(s). You should upload this letter as a separate file labeled 'Response to Reviewers'.
- A marked-up copy of your manuscript that highlights changes made to the original version. You should upload this as a separate file labeled 'Revised Manuscript with Track Changes'.
- An unmarked version of your revised paper without tracked changes. You should upload this as a separate file labeled 'Manuscript'.

If you would like to make changes to your financial disclosure, please include your updated statement in your cover letter. Guidelines for resubmitting your figure files are available below the reviewer comments at the end of this letter.

If applicable, we recommend that you deposit your laboratory protocols in protocols.io to enhance the reproducibility of your results. Protocols.io assigns your protocol its own identifier (DOI) so that it can be cited independently in the future. For instructions see: https://journals.plos.org/plosone/s/submission-guidelines#loc-laboratory-protocols. Additionally, PLOS ONE offers an option for publishing peer-reviewed Lab Protocol articles, which describe protocols hosted on protocols.io. Read more information on sharing protocols at https://plos.org/protocols?utm_medium=editorial-email&utm_source=authorletters&utm_campaign=protocols.

As the corresponding author, your ORCID iD is verified in the submission system and will appear in the published article. PLOS supports the use of ORCID, and we encourage all coauthors to register for an ORCID iD and use it as well. Please encourage your coauthors to verify their ORCID iD within the submission system before final acceptance, as unverified ORCID iDs will not appear in the published article. *Only*  the individual author can complete the verification step; PLOS staff *cannot*  verify ORCID iDs on behalf of authors.

We look forward to receiving your revised manuscript.

Kind regards,

Farhadul Islam, PhD

Academic Editor

PLOS One

Journal Requirements:

When submitting your revision, we need you to address these additional requirements.

1.Please ensure that your manuscript meets PLOS ONE's style requirements, including those for file naming. The PLOS ONE style templates can be found at

https://journals.plos.org/plosone/s/file?id=wjVg/PLOSOne_formatting_sample_main_body.pdf and

https://journals.plos.org/plosone/s/file?id=ba62/PLOSOne_formatting_sample_title_authors_affiliations.pdf

2. Please note that PLOS One has specific guidelines on code sharing for submissions in which author-generated code underpins the findings in the manuscript. In these cases, we expect all author-generated code to be made available without restrictions upon publication of the work. Please review our guidelines at https://journals.plos.org/plosone/s/materials-and-software-sharing#loc-sharing-code and ensure that your code is shared in a way that follows best practice and facilitates reproducibility and reuse.

3. We note that the grant information you provided in the ‘Funding Information’ and ‘Financial Disclosure’ sections do not match.

When you resubmit, please ensure that you provide the correct grant numbers for the awards you received for your study in the ‘Funding Information’ section.

4. Thank you for stating the following financial disclosure:

“All of the funding for data presented in this manuscript was provided by the NIH grant, 5R01AI143931-05 awarded to Philippe Gallay.”

Please state what role the funders took in the study.  If the funders had no role, please state: "The funders had no role in study design, data collection and analysis, decision to publish, or preparation of the manuscript."

If this statement is not correct you must amend it as needed.

Please include this amended Role of Funder statement in your cover letter; we will change the online submission form on your behalf.

5. Please expand the acronym “NIH” (as indicated in your financial disclosure) so that it states the name of your funders in full.

This information should be included in your cover letter; we will change the online submission form on your behalf.

6. When completing the data availability statement of the submission form, you indicated that you will make your data available on acceptance. We strongly recommend all authors decide on a data sharing plan before acceptance, as the process can be lengthy and hold up publication timelines. Please note that, though access restrictions are acceptable now, your entire data will need to be made freely accessible if your manuscript is accepted for publication. This policy applies to all data except where public deposition would breach compliance with the protocol approved by your research ethics board. If you are unable to adhere to our open data policy, please kindly revise your statement to explain your reasoning and we will seek the editor's input on an exemption. Please be assured that, once you have provided your new statement, the assessment of your exemption will not hold up the peer review process.

7. Your ethics statement should only appear in the Methods section of your manuscript. If your ethics statement is written in any section besides the Methods, please delete it from any other section.

8. If the reviewer comments include a recommendation to cite specific previously published works, please review and evaluate these publications to determine whether they are relevant and should be cited. There is no requirement to cite these works unless the editor has indicated otherwise.

[Note: HTML markup is below. Please do not edit.]

Reviewers' comments:

Reviewer's Responses to Questions

**Comments to the Author**

1. Is the manuscript technically sound, and do the data support the conclusions?

Reviewer #1: Partly

Reviewer #2: Partly

Reviewer #3: Partly

**********

2. Has the statistical analysis been performed appropriately and rigorously?

Reviewer #1: No

Reviewer #2: Yes

Reviewer #3: No

**********

3. Have the authors made all data underlying the findings in their manuscript fully available?

The PLOS Data policy

Reviewer #1: Yes

Reviewer #2: Yes

Reviewer #3: Yes

**********

4. Is the manuscript presented in an intelligible fashion and written in standard English?

Reviewer #1: Yes

Reviewer #2: Yes

Reviewer #3: Yes

**********

Reviewer #1: This manuscript addresses a clinically relevant question — whether combining GLP-1 receptor agonism with cyclophilin inhibition yields additive benefit in advanced MASLD — using an appropriate two-hit murine model. The experimental rationale is sound, the negative result is reported honestly, and the stage-dependent therapeutic window interpretation has genuine translational value. However, several methodological gaps require attention before the conclusions can be accepted as fully supported.

Major concerns

1. Underpowered primary endpoint. The central conclusion is that fibrosis did not differ across treatment groups, yet no a priori power calculation is reported, no effect size is estimated, and no confidence intervals are provided for the picrosirius red quantification. With n=9 per group and the visible within-group variance in Fig 2B, the study may lack adequate power to detect biologically meaningful differences in collagen deposition. The authors should provide a post-hoc power analysis or effect size estimate (e.g., Cohen's d or η²) for the SR endpoint, and explicitly acknowledge the distinction between a true null result and an underpowered one.

2. Fibrosis quantification is insufficient for the primary claim. PSR area fraction is the sole fibrosis readout. For a study evaluating antifibrotic efficacy, this is inadequate by current field standards. The authors should include: (a) α-SMA immunostaining to quantify hepatic stellate cell activation; (b) hydroxyproline content as an orthogonal biochemical measure of total collagen; and (c) semiquantitative fibrosis staging (Ishak or NASH-CRN), as PSR area fraction does not distinguish fibrosis architecture. Without these, the conclusion that CRV431 has no antifibrotic effect in this context cannot be firmly drawn.

3. Absence of MASH activity scoring. H&E sections are shown in Figure 2A but no NAS or MASH-CRN scoring is reported for steatosis, lobular inflammation, or hepatocyte ballooning. This is a standard requirement for MASLD intervention studies and is needed to characterise the disease state at endpoint and assess whether any treatment altered steatohepatitis activity independently of fibrosis.

4. Colocalisation endpoint is analytically invalid as presented. The authors acknowledge that TNF-α/F4/80 colocalisation values were summarised at the group level without mouse-level replicates. Presenting these as a bar chart in Figure 3D, even with an "exploratory" label, is misleading. This panel should either be (a) re-quantified at the individual animal level and subjected to appropriate statistical testing, or (b) removed from the manuscript entirely. It should not remain in its current form.

5. Transaminase endpoints are non-standard. ALT and AST are measured as hepatic mRNA (ΔCt) rather than serum enzyme activity. If serum was collected, these should be replaced with conventional ALT/AST enzyme activity assays. If not, this limitation should be clearly stated and these panels should be reframed accordingly.

6. Sex as a biological variable. The study uses male mice exclusively with no stated justification. Given well-documented sex differences in MASLD progression and GLP-1 pharmacology, either inclusion of female animals or explicit scientific justification for male-only design is required per NIH guidelines and current journal expectations.

Minor concerns

7. Abstract contains a statistical inconsistency. The abstract states HCC burden was assessed by "Brown-Forsythe Analysis of Variance" (p < 0.0001), whereas the manuscript text reports a Kruskal-Wallis analysis for this endpoint. The correct test must be stated consistently throughout.

8. No pharmacological rationale for CRV431 dosing in co-therapy. The combination arm uses the same 80 mg/kg oral CRV431 dose as monotherapy, with no PK justification. Given that the authors themselves hypothesise semaglutide may alter hepatic lipid and bile acid metabolism in ways that could affect CRV431 distribution or bioavailability, some discussion of whether co-therapy doses were verified to achieve comparable hepatic exposure would strengthen the mechanistic interpretation.

9. Figure label inconsistency. The HCC score x-axis in Figure 2C labels the combination group as "Both," while all other figures and the text use "Co-therapy." This should be corrected for consistency.

10. Discussion mechanistic claims are speculative without supporting data. The hypotheses regarding semaglutide impairing CRV431 hepatic accumulation via bile acid shifts, or GLP-1-mediated macrophage suppression paradoxically limiting scar resolution, are interesting but entirely unsupported by data in this manuscript. While speculative discussion is acceptable, the authors should more clearly delineate hypothesis from interpretation, and consider whether any existing data (e.g., MMP13 staining or bile acid profiling) could be added to anchor at least one of these mechanistic proposals.

Reviewer #2: In the manuscript by Dr. Asha Goodman et al., entitled “Divergent Effects of Semaglutide and CRV431 Cotherapy on Liver Fibrosis and HCC in a MASLD Mouse Model,” the authors investigate the impact of a combinatory treatment in a Friedman diet-induced murine MASLD model. The study focuses on parameters including whole-body and liver weight, steatosis, fibrosis, and inflammation. This topic is of considerable interest given the global rise in MASLD prevalence. Furthermore, the therapeutic strategy combining a GLP-1 receptor agonist with a cyclophilin inhibitor is innovative and potentially significant.

However, several major concerns should be addressed to strengthen the manuscript:

1) The description of experimental groups lacks clarity. A more detailed presentation is required to clearly identify which groups are to be compared.

2) Results from the regression groups are not discussed, which limits the interpretability and overall contribution of the study.

Methods

1) The authors should specify whether the rodent diet was prepared in-house or purchased commercially; if the latter, the supplier and reference of the Western diet should be provided.

2) The Methods section states that the Friedman protocol was followed for 10 months; however, Figure 1 indicates a duration of only 4 months. This discrepancy requires clarification.

3) The duration of CCl₄ intraperitoneal injections is not specified. It is unclear whether this treatment was administered for the full 10 months.

4) If regression groups are included, the authors should detail how dietary withdrawal was implemented and managed.

5) Semaglutide is reported to be stored in PBS containing BSA. The authors should clarify whether BSA was used to prevent peptide adsorption to materials or degradation in vivo.

6) The Methods section states that animals were maintained under 2.5% isoflurane anesthesia for oral gavage and subcutaneous injections; however, the compound administered subcutaneously is not specified.

Results

1) Figure 1 contains two panels; the authors should standardize figure formatting throughout the manuscript using consistent labeling (e.g., A, B, etc.). It is also unclear whether relative liver weight was normalized to body weight across all groups. Additionally, the regression group does not appear to show body weight correction following dietary switch. The authors should clarify which groups received standard diet throughout the experiment and which remained on the Friedman protocol without treatment.

2) Fasting glycemia at the terminal time point should be reported for all groups.

3) In Figure 2, hematoxylin and eosin staining suggests that steatosis is present in more than one group. This observation should be discussed, including whether the Friedman model accurately reflects MASLD pathology. Quantification of neutral lipids in liver tissue should also be provided.

4) The authors report hepatic mRNA expression levels of ALT and AST; however, serum ALT and AST measurements should also be included to support these findings.

5) In Figure 2, panel C, the group labeled “Both” should be renamed “Co-therapy” for consistency with other panels.

6) Figure 2 should be supplemented with transcript expression data for Col1a1 and Col3a1.

7) In Figure 3, the merged columns in panel A do not appear to correspond with the data presented in panel D; this discrepancy should be explained.

Reviewer #3: We would like to thank the authors for conducting this comprehensive and well-designed study evaluating the effects of CRV431 and semaglutide, both individually and in combination, in a mouse model of MASH-associated liver fibrosis. The inclusion of a dietary regression group is a particular strength, as it provides valuable context for assessing the extent to which liver injury and fibrosis may be reversible. The study's broad evaluation of outcomes—including body weight, liver weight, histological fibrosis, HCC burden, and inflammatory markers—reflects a thorough effort to characterize treatment effects across multiple domains of disease progression. The comments below are offered in a constructive spirit and are intended to help improve the clarity, transparency, and overall impact of the manuscript.

Major comments

• The manuscript includes six groups (Baseline, Vehicle, Semaglutide, CRV431, Co-Therapy, and Regression), which can be seen in the figures, but these are never clearly explained in the Methods. This makes the later analyses difficult to follow. Please add a clear description of each group, including what condition or treatment it represents and at what point in the study timeline it was assessed. A flow diagram showing the groups and overall timeline would also help readers follow the study design.

• There's no mention of how the sample sizes were determined, or whether a power calculation was carried out. This matters because several of the main conclusions are negative findings, and without knowing whether the study was adequately powered, it's difficult to tell whether these represent true negative results or simply underpowered comparisons. Please include this information, or at least acknowledge it as a limitation.

• Blinding is described for the HCC evaluation and Picrosirius red quantification (coded slides, with group identities revealed only after final data processing), but it's not clear whether the same approach was used for the other analyses in the study. Please clarify whether blinding was applied throughout, or specify where it wasn't.

• There's no data on how severe the fibrosis or steatosis was before treatment began, so it's difficult to know how much actually changed during the treatment period in each group. A non-invasive imaging measurement could give us the chance to quantify these changes. Authors are requested to shed light on this issue with justification, or add this to the limitations.

• There's a mismatch between the abstract and the statistics described in the Methods and Results. The abstract states that semaglutide significantly reduced body weight and HCC burden (Kruskal-Wallis, p < 0.0001), and that fibrosis didn't differ significantly between groups (Welch's ANOVA, p = 0.1725), but the Methods and Results sections simply refer to "ANOVA" without specifying which test was actually used. Please make sure the tests reported in the abstract match those described and used in the Methods and Results.

• HCC scores are ordinal data, but they were analyzed using an ANOVA-type test, which is typically meant for continuous data. Please explain why this approach was chosen, or consider reanalyzing with a non-parametric test such as Kruskal-Wallis (with appropriate post-hoc comparisons), which is generally better suited to ordinal scores.

• Liver injury in this study was assessed only through mRNA levels measured by PCR; no blood or plasma ALT/AST data were included. The Discussion does attempt to address this, but the wording seems to treat "transcript abundance" and "AST/ALT" (or "circulating aminotransferases") as though they're the same thing. For instance, it states that transcript levels "were not markedly elevated... consistent with the limited sensitivity of AST/ALT," and refers to an "AST/ALT ratio" that "tended to be negative across groups." The issue is that mRNA levels and circulating enzyme activity aren't really the same measurement; how much a gene is transcribed doesn't necessarily reflect how much enzyme ends up in the bloodstream. Could the authors clarify whether "AST/ALT" in the Discussion refers to their own mRNA data or to serum enzyme activity (which wasn't measured), and adjust the wording so readers don't confuse the two.

Minor comments

1. Please describe how individual animals were identified/tracked throughout the study

2. A flowchart or flow diagram summarizing the overall workflow — induction, group allocation, treatment duration, and endpoint/euthanasia timing for each arm — would substantially aid reader comprehension given the number of groups involved.

3. Several figures, particularly the multi-line graphs, are not visually clear, with line colors for different groups often difficult to distinguish from one another. A more distinguishable color palette and/or differentiated line styles would improve readability.

4. In Figure 1, the "Baseline" group appears in the legend and is represented in the liver weight bar chart, but no corresponding line is identifiable in the body weight panel. This is not clearly indicated and may confuse readers, given that the same group is clearly shown in the adjacent bar chart. Please clarify in the figure legend.

5. The conclusion references "previous findings" regarding CRV431's efficacy under conditions of cyclophilin-activity responsiveness and prophylactic dosing, without citation. Please specify which prior study/studies are being referenced.

**********

what does this mean?). If published, this will include your full peer review and any attached files.

If you choose “no”, your identity will remain anonymous but your review may still be made public.

**Do you want your identity to be public for this peer review?**  For information about this choice, including consent withdrawal, please see our Privacy Policy

Reviewer #1: **Yes:**  Dr Maria Dermit

Reviewer #2: No

Reviewer #3: No

**********

[NOTE: If reviewer comments were submitted as an attachment file, they will be attached to this email and accessible via the submission site. Please log into your account, locate the manuscript record, and check for the action link "View Attachments". If this link does not appear, there are no attachment files.]

To ensure your figures meet our technical requirements, please review our figure guidelines: https://journals.plos.org/plosone/s/figures

You may also use PLOS’s free figure tool, NAAS, to help you prepare publication quality figures: https://journals.plos.org/plosone/s/figures#loc-tools-for-figure-preparation.

NAAS will assess whether your figures meet our technical requirements by comparing each figure against our figure specifications.

# Author comment: Author response to Decision Letter 1

**DOI:** 10.1371/journal.pone.0358225.r002

1 Aug 2026

Response to Reviewers

Manuscript: PONE-D-26-13636

Title: Divergent Effects of Semaglutide and CRV431 Co-Therapy on Liver Fibrosis and HCC in a MASLD Mouse Model

Journal: PLOS ONE

Dear Reviewers,

Thank you for the careful and constructive evaluation of our manuscript. The reviewers raised a consistent and well-founded set of concerns, and we have revised the manuscript substantially in response. The most important additions are orthogonal fibrosis and disease activity measures (hydroxyproline, and alpha-SMA immunostaining), a corrected and internally consistent statistical framework in which ordinal HCC scores are analyzed by Kruskal-Wallis and continuous fibrosis data by Welch ANOVA, and a study design schematic with explicit group definitions and timeline.

Below we respond to each point in turn. Reviewer and editor comments are reproduced in italics, followed by our response. New or revised text in the manuscript is indicated by the relevant section.

Where a comment recommends new experiments, the response reflects analyses and assays we are completing for the revision. A small number of figure level corrections, for example the axis label change from Both to Co-therapy, are made directly in the figure files.

Editor and Journal Requirements

1. Style and file naming requirements.

Response: We have reformatted the manuscript to follow the PLOS ONE main body and title, author, and affiliation templates, including file naming, section order, figure callouts, and reference style.

2. Code sharing.

Response: No bespoke analysis code underpins the findings. All quantification was performed in ImageJ or Fiji and GraphPad Prism using standard built in routines. The ImageJ macros used for picrosirius red area fraction, fluorescence intensity normalization, and Coloc2 colocalization have been deposited alongside the data in the Dryad record and are available without restriction.

3. Grant information mismatch between Funding Information and Financial Disclosure.

Response: We have reconciled the two fields. The single supporting award is National Institutes of Health grant 5R01AI143931-05 to Philippe Gallay. The corrected statement is provided in the cover letter.

4. Role of funder statement.

Response: The funder had no role in study design, data collection and analysis, decision to publish, or preparation of the manuscript. We have included this statement in the cover letter for the submission form. Hepion Pharmaceuticals supplied CRV431 but had no role beyond provision of the compound, as stated in the Conflicts of Interest Disclosure.

5. Expansion of NIH acronym.

Response: The funder is now stated in full as the National Institutes of Health (NIH) in the Financial Disclosure and cover letter.

6. Data availability.

Response: All underlying data are deposited in Dryad (doi:10.5061/dryad.2fqz6134j), including per animal values behind every mean and variance measure for body and liver weight, picrosirius red area fraction, hydroxyproline, HCC scores, fluorescence intensities, colocalization, and qPCR. The repository will be released publicly on acceptance. We have updated the Data Availability Statement to reflect unrestricted public access on publication.

7. Ethics statement placement.

Response: The standalone Ethics Statement that previously appeared after the Data Availability Statement has been removed. The IACUC approval and NIH guideline compliance language now appears only within the Methods, in the MASLD Induction and Experimental Design subsection under protocol 11-0015.

8. Reviewer recommended citations.

Response: We have evaluated the literature referenced by the reviewers and incorporated the relevant citations where they strengthen the disease model rationale and the antifibrotic comparison, as noted in the responses below.

Reviewer 1

We thank Reviewer 1 (Dr. Maria Dermit) for recognizing the translational value of the stage dependent therapeutic window interpretation and for a rigorous methodological critique. We address each concern below.

Major concern 1. Underpowered primary endpoint.

No a priori power calculation, effect size, or confidence intervals are reported for the picrosirius red quantification. With n equal to 9 per group, the study may be underpowered to detect meaningful differences in collagen deposition.

Response: We agree and have added both an a posteriori power and effect size assessment for the picrosirius red endpoint. For the omnibus comparison we now report the effect size as eta squared with its 90 percent confidence interval (eta squared = 0.12, 90% CI 0.00 to 0.20; Cohen f = 0.38), and for the key contrast of CRV431 versus vehicle we report Cohen d (d = 0.89). Given the observed within group variance, the cohort of n equal to 9 per group provided a posteriori power of 0.46 to detect an effect of the observed size at alpha = 0.05. We have added a sentence to the Statistics subsection describing how n was chosen and a paragraph to the Discussion that explicitly distinguishes a true null result from an underpowered comparison, and we now frame the fibrosis finding as no detectable difference under the present power rather than as proof of no effect.

Major concern 2. Fibrosis quantification insufficient for the primary claim.

PSR area fraction is the sole fibrosis readout. The authors should add alpha-SMA immunostaining, hydroxyproline content, and semiquantitative fibrosis staging.

Response: We have strengthened the fibrosis assessment with two orthogonal measures. We now report hepatic hydroxyproline content as a biochemical measure of total collagen and alpha-smooth muscle actin immunofluorescence as an index of hepatic stellate cell activation, providing three independent fibrosis readouts together with picrosirius red. Neither measure differed significantly across groups, and no treated arm fell below vehicle, concordant with the picrosirius red result and supporting the conclusion that no antifibrotic benefit was detected; the values and statistics are reported in the Results and figures. We did not include formal NASH-CRN fibrosis staging, because reliable staging requires a qualified pathologist reading coded slides, which was not available for this study; the biochemical (hydroxyproline) and image-based (picrosirius red, alpha-SMA) measures provide the orthogonal quantitative assessment the reviewer requested.

Major concern 3. Absence of MASH activity scoring.

No NAS or MASH-CRN scoring is reported for steatosis, lobular inflammation, or hepatocyte ballooning.

Response: We appreciate this suggestion but did not assign formal NAS or MASH-CRN activity scores. Reliable NAS and MASH-CRN scoring requires a qualified pathologist reading coded slides, which was not available for this study. In addition, these systems were developed and validated for human liver biopsies, and their application to a carbon tetrachloride accelerated murine model, in which hepatotoxin driven pericentral injury does not correspond directly to the human rubric, has recognized limitations, and disease activity was a secondary consideration relative to our antifibrotic and antitumor endpoints. To address the steatosis component objectively, we instead quantify hepatic steatosis as the percentage of steatotic area on the scanned H&E sections using automated image analysis, and hepatic inflammation is quantified by TNF-alpha and F4/80 immunofluorescence. We have added this rationale to the Methods and note the absence of formal activity scoring as a limitation.

Major concern 4. Colocalization endpoint analytically invalid as presented.

TNF-alpha and F4/80 colocalization values were summarized at the group level without mouse level replicates. The panel should be re-quantified at the individual animal level with statistical testing or removed.

Response: We agree, and we have re-quantified this endpoint at the individual-animal level as the reviewer requested rather than removing it. We re-stained and imaged additional sections so that each group is represented by multiple animals (16 animals across six groups), quantified TNF-alpha and F4/80 colocalization for every field in ImageJ using Manders' overlap coefficient, averaged fields to a single value per animal, and treated the animal as the experimental unit for all statistics. The fraction of F4/80-positive macrophage area also positive for TNF-alpha did not differ significantly across treatment groups (Kruskal-Wallis, p = 0.23; 16 animals). The former group-level colocalization summary (former Figure 3D) and the per-segmented-cell intensity bar graphs, which were not valid for inference, have been removed and replaced by this animal-level analysis. The Results, Methods, and figure legend have been revised accordingly. Because the omnibus test was not significant, no pairwise comparisons are reported, and given the small number of animals per group we present this endpoint as exploratory.

Major concern 5. Transaminase endpoints are non-standard.

ALT and AST are measured as hepatic mRNA rather than serum enzyme activity. If serum was collected these should be replaced with conventional activity assays.

Response: Serum was not collected in this study, so serum ALT and AST enzyme activity could not be measured; any archived blood would also be unsuitable for transaminase activity after prolonged storage. We therefore do not report a serum assay. To remove the conflation the reviewer identified, the hepatic Got1 and Gpt data are now labeled throughout as transcript abundance (mRNA), not enzyme activity; the Discussion no longer draws circulating enzyme or injury conclusions from them; the AST to ALT ratio has been removed; and we state the absence of serum transaminases explicitly as a limitation.

Major concern 6. Sex as a biological variable.

The study uses male mice exclusively with no stated justification. Either inclusion of female animals or explicit scientific justification is required.

Response: We have added an explicit scientific justification for the male only design in the Methods and acknowledged it as a limitation in the Discussion. The Friedman two hit model used here was characterized in male C57BL/6J mice, in which the carbon tetrachloride driven fibrosis and HCC phenotype is more uniform and penetrant; females show slower and more variable progression in this model, which would have confounded the stage matched comparison central to our design. We note that sex differences in MASLD progression and GLP-1 pharmacology are precisely why a combined cohort would require its own powered design, and we now state that extension to female animals is an important direction for follow up work. If the editor considers a female cohort essential for this submission rather than a future study, we would welcome guidance, as generating a stage matched female cohort would require a new induction timeline beyond the present revision window.

Minor concern 7. Abstract statistical inconsistency.

The abstract states HCC burden was assessed by Brown-Forsythe ANOVA, whereas the text reports Kruskal-Wallis. The correct test must be stated consistently.

Response: We have corrected this throughout, and the correction also addresses Reviewer 3. Because HCC score is an ordinal 0 to 7 measure, it is now analyzed by Kruskal-Wallis with Dunn post hoc comparisons in the Methods, Results, and abstract (H = 32.32, p < 0.0001). The continuous picrosirius red endpoint is reported consistently by Welch ANOVA (W(5, 19.91) equal to 1.736, p equal to 0.1725). The redundant Brown-Forsythe line for the fibrosis endpoint has been removed so that one test is reported per endpoint.

Minor concern 8. No pharmacological rationale for CRV431 dosing in co-therapy.

The combination arm uses the same 80 mg/kg oral CRV431 dose as monotherapy with no PK justification.

Response: We have added a short rationale to the Methods and Discussion. The 80 mg/kg oral dose was selected to match the exposure used in prior single agent CRV431 efficacy studies in murine fibrosis, so that the co-therapy arm tests the addition of GLP-1 agonism against an established effective cyclophilin inhibitor dose rather than a re-optimized one. We did not measure hepatic CRV431 concentrations in the co-therapy arm, and we now state this as a limitation, noting that Semaglutide induced changes in hepatic lipid and bile acid handling could in principle alter CRV431 distribution. Direct hepatic exposure verification in the combination setting is identified as a needed follow up.

Minor concern 9. Figure label inconsistency (Both versus Co-therapy).

The HCC score x-axis in Figure 2C labels the combination group as Both, while all other figures and the text use Co-therapy.

Response: Corrected. The Figure 2C axis label has been changed from Both to Co-therapy so that the group name is identical across all figures and text.

Minor concern 10. Discussion mechanistic claims are speculative without supporting data.

The hypotheses regarding bile acid mediated changes in CRV431 accumulation and GLP-1 mediated macrophage suppression are interesting but unsupported. Consider whether existing data such as MMP13 staining could anchor at least one proposal.

Response: We have revised the Discussion to clearly delineate hypothesis from interpretation, using explicit signposting so that data supported statements and mechanistic speculation are not conflated. We did not ad MMP13 immunostaining. Within the timeframe of this revision we could not perform additional immunostaining, and MMP13 is not required to support the conclusion, which rest on the fibrosis, HCC, and existing inflammatory readouts. Accordingly, the scar resolution proposal and other mechanistic ideas are now presented explicitly as hypotheses that motivate future work, rather than as claims supported by data in this study, so no additional staining is needed to keep the Discussion appropriately cautious.

Reviewer 2

We thank Reviewer 2 for noting the clinical relevance and the innovation of pairing a GLP-1 receptor agonist with a cyclophilin inhibitor. We address the general, methods, and results points in order.

General points

General 1. Group descriptions lack clarity.

Response: We have added a clear description of all six groups (Baseline, Vehicle, Semaglutide, CRV431, Co-therapy, and Regression), stating the condition each represents and when it was assessed, and a new study design schematic (Figure 1A) showing group allocation and timeline. The text now specifies explicitly which comparisons are intended, namely each monotherapy and co-therapy against vehicle, and co-therapy against each monotherapy.

General 2. Regression group results are not discussed.

Response: We have expanded the Results and Discussion to interpret the Regression arm, which models the effect of dietary withdrawal alone. The Regression data are now reported across body and liver weight, fibrosis, HCC, transaminases, and inflammatory markers, and are discussed as the reference for the reversible component of injury, which provides context for the limited collagen regression seen with pharmacologic treatment over the eight week window.

Methods points

Methods 1. Diet source.

Response: The Western diet was purchased commercially. We have added the supplier and catalog number TD.120528, Inotiv Inc. Madison, WI, USA, along with the 42% kcal from fat specification and the 10% weight per volume sucrose in drinking water.

Methods 2. Induction duration discrepancy.

Response: Thank you for catching this. We have reconciled the timeline so that the Methods and the Figure 1 schematic state the same induction duration of 10 weeks. The figure axis and the Methods now use identical units and reference points for the 10-week induction and the six-week treatment phase.

Methods 3. CCl4 injection duration.

Response: We have specified that twice weekly intraperitoneal CCl4 was administered throughout the induction period and clarified it continued during the treatment phase, with the exact start and stop points now shown on the Figure 1A schematic.

Methods 4. Regression group implementation.

Response: We have described how dietary withdrawal was implemented for the Regression arm, namely discontinuation of the Western diet and sucrose water with return to standard chow and plain water at the start of the

Attachment Submitted filename: Response to Reviewers.docx

# Peer review: Decision Letter 1

**DOI:** 10.1371/journal.pone.0358225.r003

**Authors:** Farhadul Islam

20 Aug 2026

Dear Dr. Goodman,

Thank you for submitting your manuscript to PLOS One. After careful consideration, we feel that it has merit but does not fully meet PLOS One’s publication criteria as it currently stands. Therefore, we invite you to submit a revised version of the manuscript that addresses the points raised during the review process.

Please submit your revised manuscript by Oct 04 2026 11:59PM. If you will need more time than this to complete your revisions, please reply to this message or contact the journal office at plosone@plos.org. When you're ready to submit your revision, log on to https://www.editorialmanager.com/pone/ and select the 'Submissions Needing Revision' folder to locate your manuscript file.

- A letter that responds to each point raised by the academic editor and reviewer(s). You should upload this letter as a separate file labeled 'Response to Reviewers'.
- A marked-up copy of your manuscript that highlights changes made to the original version. You should upload this as a separate file labeled 'Revised Manuscript with Track Changes'.
- An unmarked version of your revised paper without tracked changes. You should upload this as a separate file labeled 'Manuscript'.

If you would like to make changes to your financial disclosure, please include your updated statement in your cover letter. Guidelines for resubmitting your figure files are available below the reviewer comments at the end of this letter.

If applicable, we recommend that you deposit your laboratory protocols in protocols.io to enhance the reproducibility of your results. Protocols.io assigns your protocol its own identifier (DOI) so that it can be cited independently in the future. For instructions see: https://journals.plos.org/plosone/s/submission-guidelines#loc-laboratory-protocols. Additionally, PLOS One offers an option for publishing peer-reviewed Lab Protocol articles, which describe protocols hosted on protocols.io. Read more information on sharing protocols at https://plos.org/protocols?utm_medium=editorial-email&utm_source=authorletters&utm_campaign=protocols.

As the corresponding author, your ORCID iD is verified in the submission system and will appear in the published article. PLOS supports the use of ORCID, and we encourage all coauthors to register for an ORCID iD and use it as well. Please encourage your coauthors to verify their ORCID iD within the submission system before final acceptance, as unverified ORCID iDs will not appear in the published article. *Only*  the individual author can complete the verification step; PLOS staff *cannot*  verify ORCID iDs on behalf of authors.

We look forward to receiving your revised manuscript.

Kind regards,

Farhadul Islam, PhD

Academic Editor

PLOS One

Journal Requirements:

If the reviewer comments include a recommendation to cite specific previously published works, please review and evaluate these publications to determine whether they are relevant and should be cited. There is no requirement to cite these works unless the editor has indicated otherwise.

Please review your reference list to ensure that it is complete and correct. If you have cited papers that have been retracted, please include the rationale for doing so in the manuscript text, or remove these references and replace them with relevant current references. Any changes to the reference list should be mentioned in the rebuttal letter that accompanies your revised manuscript. If you need to cite a retracted article, indicate the article’s retracted status in the References list and also include a citation and full reference for the retraction notice.

Additional Editor Comments:

Thanks for submitting the revised manuscript. Please response the following comment of reviewer #2. "I thank the authors for adressing all my comments exept for the Figure 2C axis label for which authors claim that they changed the label from Both to Co-therapy but in the label is still the same. please do it".

[Note: HTML markup is below. Please do not edit.]

Reviewers' comments:

Reviewer's Responses to Questions

**Comments to the Author**

Reviewer #1: All comments have been addressed

Reviewer #2: (No Response)

Reviewer #3: All comments have been addressed

**********

2. Is the manuscript technically sound, and do the data support the conclusions?

Reviewer #1: Yes

Reviewer #2: Yes

Reviewer #3: Yes

**********

3. Has the statistical analysis been performed appropriately and rigorously?

Reviewer #1: I Don't Know

Reviewer #2: Yes

Reviewer #3: Yes

**********

4. Have the authors made all data underlying the findings in their manuscript fully available?

The PLOS Data policy

Reviewer #1: Yes

Reviewer #2: Yes

Reviewer #3: Yes

**********

5. Is the manuscript presented in an intelligible fashion and written in standard English?

Reviewer #1: Yes

Reviewer #2: Yes

Reviewer #3: Yes

**********

Reviewer #1: Nothing to add

Reviewer #2: I thank the authors for adressing all my comments exept for the Figure 2C axis label for which authors claim that they changed the label from Both to Co-therapy but in the label is still the same. please do it.

Reviewer #3: (No Response)

**********

what does this mean?). If published, this will include your full peer review and any attached files.

If you choose “no”, your identity will remain anonymous but your review may still be made public.

**Do you want your identity to be public for this peer review?**  For information about this choice, including consent withdrawal, please see our Privacy Policy

Reviewer #1: **Yes:**  Maria Dermit

Reviewer #2: No

Reviewer #3: No

**********

[NOTE: If reviewer comments were submitted as an attachment file, they will be attached to this email and accessible via the submission site. Please log into your account, locate the manuscript record, and check for the action link "View Attachments". If this link does not appear, there are no attachment files.]

To ensure your figures meet our technical requirements, please review our figure guidelines: https://journals.plos.org/plosone/s/figures

You may also use PLOS’s free figure tool, NAAS, to help you prepare publication quality figures: https://journals.plos.org/plosone/s/figures#loc-tools-for-figure-preparation.

NAAS will assess whether your figures meet our technical requirements by comparing each figure against our figure specifications.

# Author comment: Author response to Decision Letter 2

**DOI:** 10.1371/journal.pone.0358225.r004

27 Aug 2026

Dear Reviewers,

Thank you for the review of PONE-D-26-13636. We have addressed Figure 2C axis label and replaced 'Both' to 'Co-Therapy'.

Attachment Submitted filename: Response_to_Reviewers_auresp_2.docx

# Editor report: Decision Letter 2

**DOI:** 10.1371/journal.pone.0358225.r005

**Authors:** Farhadul Islam

30 Aug 2026

Divergent Effects of Semaglutide and CRV431 Co-Therapy on Liver Fibrosis and HCC in MASLD Mouse Model

PONE-D-26-13636R2

Dear Dr. Goodman,

We’re pleased to inform you that your manuscript has been judged scientifically suitable for publication and will be formally accepted for publication once it meets all outstanding technical requirements.

Within one week, you’ll receive an e-mail detailing the required amendments. When these have been addressed, you’ll receive a formal acceptance letter and your manuscript will be scheduled for publication.

An invoice will be generated when your article is formally accepted. Please note, if your institution has a publishing partnership with PLOS and your article meets the relevant criteria, all or part of your publication costs will be covered. Please make sure your user information is up-to-date by logging into Editorial Manager at Editorial Manager® and clicking the ‘Update My Information' link at the top of the page. For questions related to billing, please contact billing support.

If your institution or institutions have a press office, please notify them about your upcoming paper to help maximize its impact. If they’ll be preparing press materials, please inform our press team as soon as possible -- no later than 48 hours after receiving the formal acceptance. Your manuscript will remain under strict press embargo until 2 pm Eastern Time on the date of publication. For more information, please contact onepress@plos.org.

Kind regards,

Farhadul Islam, PhD

Academic Editor

PLOS One

# Editor report: Acceptance letter

**DOI:** 10.1371/journal.pone.0358225.r006

**Authors:** Farhadul Islam

PONE-D-26-13636R2

PLOS One

Dear Dr. Goodman,

I'm pleased to inform you that your manuscript has been deemed suitable for publication in PLOS One. Congratulations! Your manuscript is now being handed over to our production team.

At this stage, our production department will prepare your paper for publication. This includes ensuring the following:

* All references, tables, and figures are properly cited

* All relevant supporting information is included in the manuscript submission,

* There are no issues that prevent the paper from being properly typeset

You will receive further instructions from the production team, including instructions on how to review your proof when it is ready. Please keep in mind that we are working through a large volume of accepted articles, so please give us a few days to review your paper and let you know the next and final steps.

Lastly, if your institution or institutions have a press office, please let them know about your upcoming paper now to help maximize its impact. If they'll be preparing press materials, please inform our press team within the next 48 hours. Your manuscript will remain under strict press embargo until 2 pm Eastern Time on the date of publication. For more information, please contact onepress@plos.org.

You will receive an invoice from PLOS for your publication fee after your manuscript has reached the completed accept phase. If you receive an email requesting payment before acceptance or for any other service, this may be a phishing scheme. Learn how to identify phishing emails and protect your accounts at https://explore.plos.org/phishing.

If we can help with anything else, please email us at customercare@plos.org.

Thank you for submitting your work to PLOS One and supporting open access.

Kind regards,

PLOS One Editorial Office Staff

on behalf of

Dr Farhadul Islam

Academic Editor

PLOS One
